# Ectopic expression of *Arabidopsis Target of Rapamycin (AtTOR)* improves water-use efficiency and yield potential in rice

**DOI:** 10.1038/srep42835

**Published:** 2017-02-23

**Authors:** Achala Bakshi, Mazahar Moin, M. Udaya Kumar, Aramati Bindu Madhava Reddy, Maozhi Ren, Raju Datla, E. A. Siddiq, P. B. Kirti

**Affiliations:** 1 Department of Plant Sciences, University of Hyderabad, Hyderabad-500046, India; 2 Department of Crop Physiology, University of Agricultural Sciences-GKVK, Hebbal, Bangalore, India; 3 Department of Animal Biology, University of Hyderabad, Hyderabad-500046, India; 4 Plant Biotechnology Institute, National Research Council of Canada, Saskatoon, Saskatchewan, S7N 0W9, Canada; 5 Biotechnology research Institute, Chinese academy of Agricultural Sciences, Beijing, P.R. China; School of Life Sciences, Chongqing University, Chongqing, P.R. China; 6 Institute of Agricultural Biotechnology, PJTS Agricultural University, Rajendranagar, Hyderabad-500030, India

## Abstract

The target of Rapamycin (TOR) present in all eukaryotes is a multifunctional protein, regulating growth, development, protein translation, ribosome biogenesis, nutrient, and energy signaling. In the present study, ectopic expression of *TOR* gene of *Arabidopsis thaliana* in a widely cultivated *indica* rice resulted in enhanced plant growth under water-limiting conditions conferring agronomically important water-use efficiency (WUE) trait. The *AtTOR* high expression lines of rice exhibited profuse tillering, increased panicle length, increased plant height, high photosynthetic efficiency, chlorophyll content and low ∆^13^C. Δ^13^C, which is inversely related to high WUE, was as low as 17‰ in two *AtTOR* high expression lines. These lines were also insensitive to the ABA-mediated inhibition of seed germination. The significant upregulation of 15 stress-specific genes in high expression lines indicates their contribution to abiotic stress tolerance. The constitutive expression of *AtTOR* is also associated with significant transcriptional upregulation of putative TOR complex-1 components, *OsRaptor* and *OsLST8*. Glucose-mediated transcriptional activation of *AtTOR* gene enhanced lateral root formation. Taken together, our findings indicate that *TOR*, in addition to its multiple cellular functions, also plays an important role in response to abiotic stress and potentially enhances WUE and yield related attributes.

Rice is a staple cereal food consumed by more than half of the world population and widely cultivated mostly in Asian countries. Environmental factors such as drought and high salinity are the major yield constraints for the high productivity of rice. The recent advancements in genetic engineering has provided new opportunities to enhance yield potential and tolerance to drought and other stresses by manipulating some of the regulatory genes involved in complex physiological and biochemical processes such as osmotic adjustment under water deficit environment.

The Target of Rapamycin (*TOR*) is one of such genes, which encodes a protein that is involved in the regulation of diverse cellular and metabolic processes, distributed in all eukaryotes including plants, yeast, animals and human. It has important functions in growth, development and metabolism. TOR is a conserved 280 kDa serine/threonine protein kinase belonging to a phosphatidylinositol-3-kinase family possessing five conserved domains represented by HEAT repeat, FAT (Focal adhesive Target), FRB (FKBP12, FK506 drug binding protein 12/Rapamycin Binding), Kinase and FATC domains from N to C-terminus, respectively. HEAT repeats bind directly to the promoter and 5′ UTR regions of 45S rRNA via its Leucine zipper domain and regulates 45S rRNA synthesis^1^. Inducible *TOR* knockout Arabidopsis plants displayed growth arrest via a reduction in polysome accumulation, which ultimately resulted in a severe reduction in plant biomass, organ and cell size^2^. FAT domain is involved in protein-protein interactions, while FRB is sensitive and specifically binds to an anti-inflammatory drug, Rapamycin^3^,^4^,^5^,^6^. The Kinase domain belongs to Ser/Thr kinase family that regulates embryogenesis and nuclear localization. Deletion of kinase domain in Arabidopsis resulted in embryonic lethality^1^. The FATC at the C-terminal region is involved in protein scaffolding.

Yeast expresses two TOR proteins; TOR1 and TOR2, whereas animals and plants possess only one copy of TOR. In yeast and mammals, TOR exists as two complexes TORC1 and TORC2 whereas plants have only one known TORC complex. These TOR complexes are mainly localized to endo-membranes and the nucleus and also exhibit mobilization according to the varied environmental conditions^7^. The TORC1 complex composed of TOR proteins, LST8 and Raptor^6^ (Regulatory associated protein of TOR) regulates translation, nutrient and energy signaling and is sensitive to Rapamycin^6^. The Rapamycin-insensitive TORC2 complex consists of TOR, Rictor (Rapamycin-insensitive companion of TOR), Lst8 and SIN1^8^. However, these TORC2 components have not been identified so far in plants and other photosynthetic organisms^5^,^6^,^9^,^10^. TORC2 regulates the organization of cytoskeletal structure and cell survival^6^,^11^.

Unlike animals and yeast, plant TOR is insensitive to rapamycin^12^,^13^,^14^. Based on the position of introns in the genic regions of TORC1 components, Raptor and LST8 in human are similar to plants indicating that they are evolutionarily conserved^10^. The glucose-activated TOR signaling regulates genes involved in root meristem growth, cell proliferation, cell cycle, and nucleotide synthesis^12^. Auxin-activated TOR pathway regulates translation initiation and polysome accumulation^15^. Other phytohormones such as ABA regulate plants response to abiotic stresses such as dehydration and salinity by controlling stomatal aperture and transcription of stress-responsive genes^5^,^16^ while other *cis-*regulating elements such as DREB1/CBF and DREB2A are ABA-independent dehydration stress-responsive elements.

The multiple metabolic and regulatory cellular functions of *TOR* and also presence of *cis*-regulatory elements in the putative promoter region of its gene have prompted us to study the effect of ectopic expression of *AtTOR* for its ability to enhance water-use efficiency (WUE) in rice without compromising on the yield, which is a very important manipulation with significant agronomic considerations. We have generated transgenic rice plants overexpressing *AtTOR* and our key findings in the context of WUE, phenotypic and physiological characterization and regulation of stress-specific genes in high *TOR* expression lines in a very widely cultivated *indica* variety of rice, BPT-5204 (Samba Mahsuri), in which water is a major yield limiting factor, are reported here.

## Materials and Methods

### Regulation of *OsTOR* by Amino acid supplements

Amino acids induce the expression of *TOR* gene in mammalian systems. To check whether the plant TOR is also regulated by amino acids, wild type (WT) *indica* rice seedlings were germinated on 15 different amino acid supplements at a concentration of 1 mM each on solid MS medium. The WT seedlings grown on MS medium without amino acid supplement were taken as a control to normalize the expression patterns. The shoot and root samples were collected after 7 d of treatment. Total RNA was isolated from root and shoot tissues, and the synthesized cDNA samples were used for qRT-PCR analysis.

### Germination of WT rice seeds on Rapamycin

TOR protein exists as two complexes, TORC1 and TORC2. The TORC2 is insensitive to Rapamycin. The structural analysis of yeast TORC2 complex showed that it has a pseudo-symmetrical rhomboid shape with the prominent central cavity and its C-terminal subunit, Avo3 resembles FRB domain of TOR2 protein. Thus, a Rapamycin-sensitive, TORC2 can be produced by removing the C-terminal sequences^17^. Plants have only TORC1 complex, which is insensitive to Rapamycin. Arabidopsis was insensitive even at concentrations >10 μg/mL Rapamycin^14^. In contrast to this, maize was found to be sensitive to even 0.1 μM Rapamycin^18^. To check the sensitivity of rice to Rapamycin, WT rice seeds were allowed to germinate after surface sterilization on solid MS medium supplemented with six different concentrations of Rapamycin separately *viz*., 20 μg/mL, 50 μg/mL, 100 μg/mL 150 μg/mL, 200 μg/mL and 250 μg/mL. Seed germination was compared with WT seeds cultured on solid MS medium without Rapamycin.

### Protein extraction and Western blot Analysis

Total protein was extracted from one gram of 200 μg/mL and 250 μg/mL Rapamycin-treated and untreated 7 d-old WT seedlings by phenol extraction method. The protein precipitate was re-suspended in rehydration buffer (7 M urea, 2 M thiourea, 4% CHAPS and 30 mM DTT) and quantified by Bradford method using BSA as a standard. About 60 μg of total protein was loaded for SDS-PAGE and Western blot analysis.

To detect the TOR-mediated phosphorylation of OsS6K1, Western blot was performed using mouse antibody against human phospho70S6K1 (Thr(P)-389) (Cell Signaling Technologies, cat# 9206), anti-70S6K1 (CST, cat# 2708S), anti-GAPDH (Santa Cruz, FL-335#SC25778) with respective HRP-conjugated secondary antibodies and immunoreactions were detected by a chemiluminescent method (ChemiDoc XRS, Bio-Rad). S6K1 proteins of human, Arabidopsis and rice are 70 kDa proteins and the phosphorylation sites of these proteins are conserved. The human S6K1 antibodies were demonstrated to be reactive against S6K1 proteins of plants such as Arabidopsis and rice^12^,^13^,^19^. Because of the high conservation and specificity, we have used human p70S6K1 antibody raised in mouse (CST, cat# 9206) to detect rice S6K1 phosphorylation.

### *AtTOR* Vector details and *in planta* transformation of *indica* rice

The 7.4 kb, full-length *TOR* cDNA was derived from *Arabidopsis thaliana* (Col.). The cDNA was amplified using *Not*I restriction site at the 5′ end and *Xmal*I site at the 3′ end of the sequence. The full-length *AtTOR* was then cloned into the p8GWN vector at *Not*I/*Xma*I sites and was finally transformed into binary vector, pEarleyGate 203 through LR recombination. The binary vector also carries the *bar* gene under Mannopine synthase promoter as a plant selection marker^1^.

The binary vector carrying 35S: *At*TOR was transformed into a widely cultivated *indica* rice variety BPT-5204 using a novel *in planta* transformation protocol. Rice seeds of BPT-5204 (Samba Mahsuri) were manually de-hulled and surface sterilized with 70% ethanol for 1 min and then twice for 10 min with 4% aqueous sodium hypochlorite containing a drop of Tween-20, followed by five washes with sterile double-distilled water. Sodium hypochlorite not only sterilizes the seeds but also removes the seed waxes, which could otherwise inhibit the entry of *Agrobacterium*^20^. The *Agrobacterium* culture carrying the binary vector, 35S: *AtTOR* was grown overnight at 28 °C on a rotary shaker in 100 mL Luria Bertani (LB) medium containing the antibiotics Kanamycin and Rifampicin (50 mg/L each). The culture (OD_600_ = 1.0) was centrifuged at 5000 rpm for 5 min and the pellet was re-suspended in an equal volume of *Agrobacterium* suspension solution (MS basal salts supplemented with 68 g/L sucrose, 10 g/L glucose, 0.6 g/L proline, 200 μM Acetosyringone, 3g/L each of KCl and MgCl_2_ at pH 5.4). A fine sterile needle was dipped in the *Agrobacterium* suspension and gently pierced at the basal part of the embryo of seeds. After infection, the infected seeds were kept in a vacuum jar. Using an air-out vacuum system, air was evacuated and the seeds were maintained under a vacuum of about 15 mmHg for 20 min, after which the vacuum was released as quickly as possible. Following this, the infected seeds were further incubated in the *Agrobacterium* suspension at 28 °C under gentle agitation (100 rpm) for 20 min. Then the seeds were maintained for 3 d in the dark at 28 °C with the embryonic portion of the seed in contact with the co-cultivation medium (MS salts containing 30 g/L sucrose, 1.0 g/L proline, 300 mg/L casein hydrolysate, 150 μM Acetosyringone and 7 g/L agar, pH 5.4). After 3 d, seeds were washed thrice with water and allowed to recover on blotting paper moistened with half strength liquid MS medium^21^ containing the antibiotics, Carbenicillin and Cefotaxime (250 mg/L each) up to a week until the growth of the seedlings was observed. The seedlings were then transferred to pots and grown under greenhouse conditions to select for T_1_ plants.

### Selection and molecular analysis of rice *AtTOR* transgenic plants

The T-DNA of the *AtTOR* binary vector contains a plant selection marker gene, *bar* that provides resistance to the herbicide phosphinothricin (PPT). Seeds obtained from the *Agrobacterium* treated rice plants were germinated on a medium containing 10 mg/L PPT to select the transgenic plants for a detailed analysis and maintenance. Those seeds that germinated on the PPT selection medium were transferred to the pots in the greenhouse under the controlled conditions.

PCR amplification was performed on the transformed plants using primers specific for the various elements present in the T-DNA. PCR reactions were performed in a 25 μl total reaction volume containing 2.5 μl 10X buffer, 1 unit *Taq* polymerase, 10 μM of each primer and 10 ng of plasmid (35S: *At*TOR) was used as a positive control. PCR was carried with an initial denaturation at 94 °C for 4 min followed by 35 repeated cycles of 94 °C for 1 min, 56 °C for 50 sec, and 72 °C for 1 min. This was followed by a final extension at 72 °C for 10 min. After PCR confirmation, seeds were collected from T_1_ generation plants and were proceeded further to raise T_2_ generation by screening them again on the selection medium. All the phenotypic, physiological and molecular analysis were carried out on T_2_ generation PPT resistant transgenic plants (which are a mixture of homozygous and hemizygous plants). The observations on the responses of ABA and glucose and the expression studies of stress-related genes and TORC1 complex components have been obtained from T3 generation plants.

### Southern-blot hybridization analysis of transgenic plants

Southern-blot hybridization was performed to confirm the transgenic nature of the plants and also to verify the copy number of the integrated T-DNA in the genomes of transgenic rice plants. Genomic DNA was isolated from the leaves of T_2_ generation plants using the CTAB method. About 10 μg of genomic DNA was digested overnight with *Asi*SI and *Cla*I restriction enzymes (which does not have internal restriction sites within the T-DNA) at 37 °C. The fully digested genomic DNA fragments were electrophoresed on 0.8% agarose gel at 20 V for 16–18 h. The digested DNA fragments were alkali (NaOH) denatured and transferred onto Hybond N^+^ nylon membrane (GE Healthcare Life Sciences), followed by UV cross-linking (120 kJ/cm^2^). The PCR-amplified product of the *bar* gene was labeled with DIG-dUTP using the Klenow polymerase and used as a probe in the hybridization analysis. After hybridization and repeated stringency washes, binding of the probe was detected using anti-DIG-alkaline phosphatase enzyme and NBT/BCIP substrate according to the manufacturer’s protocol (Roche Life Sciences, Germany).

### Semi-quantitative (semi-Q) and Quantitative real-time PCR (qRT-PCR)

Semi-Q and qRT-PCR analysis were performed on T_2_ generation rice transgenic plants to separate the low, medium and high expression lines of *AtTOR*. Total RNA was isolated from the leaves of one-month-old transgenic and WT plants using Trizol (Sigma-Aldrich, US) method. About 2 μg of total RNA was used for first-strand cDNA synthesis using SMART^TM^ MMLV Reverse Transcriptase (Takara Bio, Clontech, USA). The synthesized cDNA was diluted seven times (1:7) and 2 μl of this was used for analyzing the transcript level of *TOR* gene using primers that specifically bind and amplify *Arabidopsis* TOR-kinase. The conditions used in semi-Q PCR include an initial denaturation at 94 °C for 3 min, followed by 26 repeated cycles of 94 °C for 30 sec, 61 °C for 25 sec and 72 °C for 30 sec with a final extension for 5 min at 72 °C. The *OsActin* was used as an endogenous reference gene in both semi-Q and qRT-PCR analyses of all the genes studied. Based on the band intensity observed on the agarose gel after semi-Q PCR, the transgenic lines were categorized as low, medium and high expression lines, which were further characterized with qRT-PCR.

The same cDNA that was diluted in 1:7 proportions used in semi-Q PCR was also used in qRT-PCR to analyze the transcript levels in three classes of *At*TOR plants (low, medium and high) that were separated through semi-Q PCR. Since the *TOR* gene in rice transgenic plants has been derived from Arabidopsis, WT rice cannot be used to normalize the expression. Therefore, we have used a low-expression transgenic plant as a control to normalize the expression using primers specific to *AtTOR*. Among these, the transgenic plant, TR5.1 with a weak band intensity (low-expression line) in Semi-Q PCR was considered as a control to normalize the fold level in qRT-PCR, performed using SYBR Green ^®^ Premix (Takara Bio, USA). The qRT-PCR reaction conditions included an initial denaturation at 94 °C for 2 min, followed by 40 cycles of 94 °C for 15 sec, 61.9 °C for 25 sec and 72 °C for 30 sec followed by a melting curve. The qRT-PCR data was analyzed according to the ΔΔC_T_ method^22^.

### Transcript analysis of *OsTOR* Complex-1 (TORC1) components in transgenic rice plants

The OsRaptor and OsLst8 are the components of TORC1 complex that directly interact with HEAT repeats and the kinase domain of TOR protein, respectively. To check whether the expression pattern of these two genes varied in relation to the expression of *AtTOR* in rice transgenic plants, we analyzed the transcript levels of rice *OsRaptor* and *OsLst8* in root and shoot tissues of two medium (TR10.2 and TR22.6), two high (TR2.24, TR15.10) and one low *AtTOR* expression lines (TR5.1) in 7 d old seedlings through qRT-PCR.

### Screening of transgenic plants for high WUE

To examine whether *TOR* enhances WUE in rice, the T_2_ generation *AtTOR* transgenic plants along with WT were grown under limited water availability conditions. After selection on PPT medium for two weeks, the *AtTOR* transgenic plants along with WT were transferred to pots and allowed to grow with adequate water supply in the pots (with 3–4 cm water overlay as in normal conditions) in the greenhouse. After four weeks of normal growth, the surface water was withdrawn, and the water level was adjusted such that wet/moist conditions were maintained in the soil (100% field capacity). These conditions were maintained till the plants reached maturity. All the phenotypic and physiological observations were recorded during these growth conditions of the plants, and the measurements were compared with two types of WT, one grown with adequate water supply and the other maintained along with transgenic plants under water-limited conditions.

### Phenotypic characterization of transgenic plants

Yield-related phenotypic parameters such as the number of tillers, productive tillers (panicles), panicle length and plant height were recorded 45 d after growth under limited water conditions and 70 d after transfer to the greenhouse. The phenotypic parameters were collected from five individual plants of each transgenic line and WT, and the mean of these recordings was plotted as a histogram using an online tool (http://www.wessa.net/).

### Measurement of Chlorophyll fluorescence of photosystem-II

Chlorophyll (Chl) fluorescence was measured using a portable pulse-amplitude modulated photosynthesis yield analyzer (MINI-PAM) essentially according to the manufacturer’s protocol (Heinz Walz, Germany). Chl fluorescence is a key indicator of the overall photosynthetic performance of a plant and also indicates whether a plant is experiencing stress^23^. It also gives an insight into the use of excitation energy by photosystem II (PSII).

When leaves, that have been adapted to a brief dark period are exposed to a beam of low intensity light (0.1 μmol m^2 ^s^1^), chlorophyll gets excited to a minimal level (*F*_*o*_). Application of a saturating pulse (8000 μmol m^2 ^s^1^) results in the formation of a maximum possible yield of fluorescence (*F*_*m*_). The difference between *F*_*m*_ and *F*_*0*_gives a variability in fluorescence (*F*_*v*_) and the effective quantum yield of PSII was calculated as *F*_*v*_/*F*_*m*_^23^. The quantum efficiency of unstressed plants grown under normal conditions was in the range of 0.83–0.84. Low *F*_*v*_/*F*_*m*_ values indicate that plants are under stress; higher values represent high quantum yield^24^. Two readings were taken in all the transgenic plants along with WT after exposing them to a dark period of 30 min at an interval of 15 d each after withdrawing water. The mean of all these readings was plotted as a histogram.

### Estimation of Chlorophyll content

The Chl content was measured in the leaves of WT, low, medium and high expression lines three weeks before and after their growth under water deficit conditions. The Chl was extracted using 100 mg of leaf tissue in 80% acetone and absorption of the extracts was measured at OD 663 nm and 645 nm using a UV spectrophotometer^25^. The concentrations of Chl-a, Chl-b and total chlorophyll (Chl-t) were then calculated using the equations as described earlier^26^.

### Δ^13^C analysis of transgenic plants

Carbon exists as two stable isotopes (^13^C and ^12^C) in the atmosphere with a molar ratio of 1:99, C_3_ photosynthetic plants prefer ^12^C over ^13^C^27^. The photosynthetic enzyme RubisCO discriminates the carbon isotopes during photosynthesis by sensing the difference in CO_2_ diffusion through stomata. Environmental factors such as temperature, water and other stresses reduce the leaf stomatal conductance due to which the internal leaf CO_2_ concentration gradient also changes, which ultimately affects carbon assimilation during photosynthesis in plants. These changes due to environmental effects can be assessed by carbon isotope composition (Δ^13^C) in leaf tissue i.e. discrimination against heavier isotope (^13^C) from lighter isotope (^12^C) in C_3_ or C_4_ plants during photosynthesis. Deviation in CO_2_ concentration, changes the biochemical and metabolic products and contributes to variation in plants internal isotopic composition. This deviation from the existing ratio can be used to know the internal changes in plants due to the environmental stress factors. This ratio of ^13^C/^12^C can be correlated to the ratio of photosynthesis and stomatal conductance, which is also known as water-use efficiency (WUE).

Under water limited conditions stomata tend to close to reduce the transpirational water loss, which in some genotypes results in an improvement of the WUE. During such situations, plants exhibit low internal CO_2_ concentration and reduced discrimination for ^13^C from ^12^C along with improved WUE. This increase in WUE results in decreased biomass of the plant. The Δ^13^C value calculated as a molar ratio (R) of ^13^C/^12^C of transgenic plants is compared to WT (R sample leaf/R standard control leaf –1) ‰ and is normalized with the international PDB standard, which has a deviation of approximately 8‰ for free atmospheric CO_2_^27^. To measure Δ^13^C and hence WUE, 150 mg of leaf samples of transgenic plants and WT grown under limited water conditions for 45 d were dried at 65 °C for three days. The powdered samples were analyzed in Isotope Ratio Mass Spectrometer (IRMS) to determine the carbon isotope ratios^28^.

### Determination of Glucose-mediated activation of TOR

Previous reports have suggested that Glucose-mediated activation of TOR is necessary for root meristem growth^12^. The seeds of low, medium and high expression lines along with WT were germinated on MS medium with and without glucose (0.3% w/v). The growth of primary and lateral roots in transgenic lines and WT grown with or without glucose was observed 5 d after germination (DAG) under a stereomicroscope. The lateral roots were counted from five seedlings of each treated and untreated samples and mean values were plotted as bar diagrams with ± standard error. On 7 DAG, RNA was extracted from WT and each transgenic line and the expression of the kinase domain of *TOR* gene was determined in transgenic lines (T_3_) grown with and without glucose, which were normalized with WT seedlings that were grown with and without glucose, respectively.

### Seed germination assays on ABA

About 25 seeds from each of low, medium and high *AtTOR* rice expression lines along with WT were allowed to germinate on MS medium supplemented with ABA (50 μM). The seed germination rate was scored at 3 and 5 DAG. All the experiments were repeated thrice and the average of three replicates was plotted with standard errors.

### Stress treatments and transcriptional regulation of stress-specific genes in high expression lines of *AtTOR* rice transgenic plants

To investigate whether TOR regulates the expression of any stress-specific genes, we analyzed the expression pattern of 15 stress-specific genes in both abiotic stress treated and untreated seedlings of T_3_ generation rice *AtTOR* high expression lines through qRT-PCR. The transcript levels of stress-specific genes in untreated seedlings of high expression lines were normalized with untreated WT seedlings while the transcript levels in treated seedlings of high expression lines were normalized with abiotic treated seedlings of WT. The two high expression lines, TR2.24 and TR15.10 having single T-DNA integration copy, high photosynthetic yield, and low Δ^13^C were selected for the expression studies. Seeds derived from these lines carrying *AtTOR* along with WT were germinated on MS medium and grown for two weeks at 28 °C, 12 h light-dark photoperiod. After two weeks, seedlings of the two transgenic lines along with the WT were subjected to three abiotic treatments such as ABA (100 μM), 15% (w/v) PEG-8000 and NaCl (300 mM). After treatments root and shoot samples of transgenic and WT samples were collected separately as three biological replicates at 5 min, 3 h, 6 h and 24 h intervals after treatments. Total RNA isolation and cDNA synthesis was performed as discussed earlier. The cDNA was diluted as 1:7 proportions and 2 μl of diluted cDNA was used to analyze the transcript level of each gene by qRT-PCR. The *OsActin* was used as an endogenous reference gene and fold changes of transgenic lines were normalized using seedlings dipped in water at corresponding time intervals. Each qRT-PCR reaction was performed as three technical replicates using samples collected in three biological replicates. Fifteen stress-specific genes from rice such as *OsDHODH1, OsNADPH1, OsSKIP1a, OsALDH2a, OsAOX1a, OsTPP1, OsLEA3-1, OsGL1-2 OsbZIP23, OsNAC1, OsNAC2, OsWRKY72, OsDREB2B, OsPP2C* and *OsSIK1* that were shown to be involved in earlier studies on immediate response in plants to abiotic stress were selected and their expression was analyzed in shoot and root tissues of untreated and ABA, PEG and NaCl-treated *AtTOR* high expression lines.

### *In silico* putative promoter and protein phylogenetic analysis of TOR

To identify the presence of *cis-*regulatory sequences in the putative promoter regions of rice and Arabidopsis *TOR* genes, a 1.5 kb sequence upstream of each gene was obtained from OryGenesDB and TAIR databases, respectively and submitted in PlantCare database. To analyze the protein phylogeny, protein sequences of TOR derived from the members belonging to Poaceae (*Sorghum bicolor; SbTOR, Zea mays; ZmTOR, Triticum uratu; TuTOR, Oryza sativa* ssp. *japonica, O. sativa* ssp. *indica, O. sativa* ssp. *brachyantha*), Brassicaceae (*A. thaliana; AtTOR, Brassica napus;* BnTOR), Solanaceae (*Solanum tuberosum; StTOR, Nicotiana tabacum*), Fabaceae (*Vigna radiate; VrTOR, Arachis duranensis; AdTOR*), Malvaceae (*Gossypium rainmondii*) and Pedaliaceae (*Sesamum indicum)* were aligned with TOR sequences of human (HumanTOR), *Caenorhabditis elegans (CeTOR*), *Drosophila melanogaster (DmTOR*), *Saccharomyces cerevisiae* (ScTOR1 and ScTOR2 aligned sequence) and *Chlamydomonas reinhardtii (CrTOR*) that were retrieved from NCBI. The retrieved sequences were aligned and the protein-based phylogenetic tree was constructed using MEGA7 program.

## Results

### Activation of *OsTOR* by amino acids

The predicted OsTOR is a 270 kDa protein composed of 2465 amino acids with a p*I* of 6.62. Amino acid residues such as leucine, alanine and serine are widely distributed constituting 12.8%, 9.2% and 7.3% of the total OsTOR protein, respectively. In shoots, arginine, leucine, and proline upregulated the *OsTOR* expression above 8-fold while serine and methionine-induced the transcript level up to 3-fold (Supplementary Fig. 1a). In roots, arginine, leucine, and proline activated the *OsTOR* transcript level more than a 10-fold while, alanine, valine, glutamine, threonine, isoleucine and phenylalanine-induced the expression up to 3-fold (Supplementary Fig. 1b). The *OsTOR* expression was downregulated by cysteine, tryptophan, histidine and lysine in both shoots and roots. Leucine, an essential component for TOR activation transports into the cells through glutamine-dependant manner and activates mTORC1 by phosphorylation of serine2448 residue through the Rag-GTPase pathway^29^,^30^,^31^.

### Rapamycin insensitivity of WT rice

TOR protein belongs to a phosphatidylinositol-3-kinase related kinase family, and it is a target of an anti-proliferative drug, Rapamycin^4^. The formation of a ternary complex of Rapamycin binding FRB (FKBP12/rapamycin- binding) domain of TOR with FK506-binding protein 12 leads to the inactivation of TOR protein where Rapamycin forms non-covalent links between FRB domain and FKBP12 protein^32^. In contrast to *Drosophila*, mammals and yeast, plant TOR was found to be insensitive to Rapamycin. Arabidopsis vegetative growth was not inhibited by Rapamycin even above 10 μg l^−1^ concentration, whereas the growth of yeast was restricted at even 0.1 μg l^−1^ of Rapamycin^14^,^33^,^34^. This insensitivity might be due to the inability to form the FKBP12–Rapamycin–FRB complex in the plants^34^. To check the sensitivity of OsTOR to the drug, Rapamycin, we germinated WT rice seeds on six different concentrations of the antibiotic, 20 μg/mL, 50 μg/mL, 100 μg/mL, 150 μg/mL, 200 μg/mL and 250 μg/mL. At as high as 250 μg/mL Rapamycin concentration, there was no inhibition of the germination and growth of rice seeds, indicating that OsTOR is also insensitive to Rapamycin (Supplementary Fig. 1c).

### Rapamycin insensitivity of *OsTOR*-kinase

**Figure 1:**
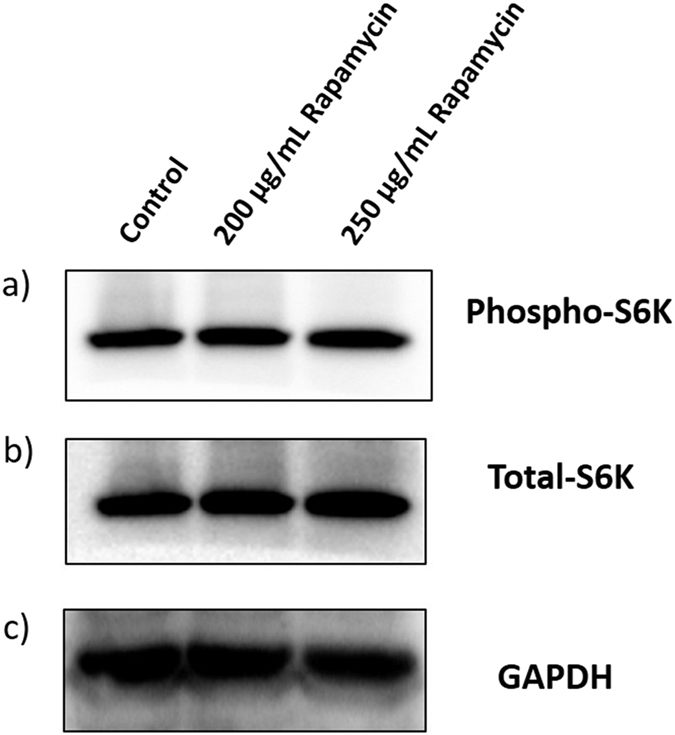
Western blot analysis to determine effect of rapamycin on OsS6K phosphorylation. Western blot analysis was performed with 7d-old WT untreated and 200 μg/mL and 250 μg/mL Rapamycin-treated seedlings using (**a**) Human anti-phospho-p70S6K (Thr(P)-389), (**b**) anti-70S6K, and (**c**) anti-GAPDH with respective HRP-conjugated secondary antibodies. No inhibition of TOR-mediated-S6K phosphorylation was observed in Rapamycin-treated WT rice seedlings.

Rapamycin blocks TOR mediated phosphorylation of S6K in eukaryotic cells. Thus TOR-kinase activity based on S6K phosphorylation had been used as a tool to unravel the TOR downstream signaling pathways in plants. Since no inhibition was observed in seed germination even at higher concentrations of Rapamycin, we checked the TOR-kinase activity based on OsS6K phosphorylation by using anti-mouse-phospho-p70S6K (Thr(P)-389). Although the inhibition of TOR kinase activity had been reported with Rapamycin treatment in maize^18^, S6K phosphorylation was detected in our study in rice seedlings germinated even at high concentrations (200 and 250 μg/mL) of Rapamycin indicating insensitivity of OsTOR kinase activity by Rapamycin (Fig. 1). Since, human S6K1 antibody has been shown to detect the phosphorylation of S6K1 in plant systems like Arabidopsis and rice^12^,^13^,^19^, we have used the same in our analysis and the binding appears to be very specific. The full Western blots and protein gel equal loading were depicted in Supplementary Fig. 1d. As, it has been shown that the activation of S6K1 phosphorylation would be undertaken by TOR-Raptor2 complex in rice^19^, we have also demonstrated the activation of OsRaptor in the AtTOR transgenic lines of rice, implying that rice S6K1 would also get activated by the same complex.

### Screening of transformants and molecular analysis of *AtTOR* rice transgenic plants

**Figure 2:**
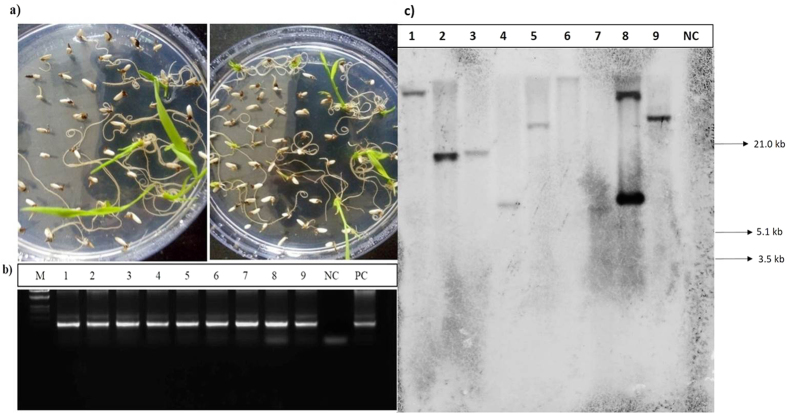
Selection and Molecular screening of transgenic plants. (**a**) Germination of primary transformed 35 S: *At*TOR seeds on PPT (10 mg/L) selection medium. PCR screening of transformants selected on PPT medium with (**b**) *bar* gene (550 bp) in T_1_ generation. M, λ.*Eco*RI-*Hin*dIII DNA Marker; PC, Positive Control; NC, Negative Control, 1.9 transgenic plants. (**c**) Southern-blot hybridization analysis of T_2_ generation 35 S: *At*TOR (Lanes 1–10 represents line numbers TR2.24, TR15.10, TR10.2, TR22.6, TR5.1, TR17.6, TR21.1, respectively) plants were digested with the restriction enzymes *AsiS*I and *Cla*I and hybridized with the DIG-dUTP labeled *bar* gene probe.

The binary vector contains the *bar* gene as a selection marker, conferring resistance to the herbicide phosphinothricin. Seeds obtained from the primary *Agrobacterium*-treated plants were screened on PPT selection medium. The transgenic seedlings started germination within 3–4 d after inoculation, while non-transgenic and WT became bleached (Fig. 2a). After selection, plants were analyzed for the different elements present in the T-DNA by PCR amplification (Fig. 2b). The *Agrobacterium* transformation rate was calculated in T_1_ generation as described by Clough and Bent, 1989^35^:


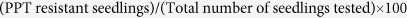


Of about 500 primary plants that were infected with *Agrobacterium* carrying *AtTOR* binary vector, 127 were found to be positive on 10 mg/L PPT selection medium with a transformation efficiency of 25.4%.

### Southern-blot hybridization analysis

Southern-blot hybridization was performed to confirm the transgenic nature and also to investigate the copy number of T-DNA integration into the genome of the T_2_ generation rice transgenic plants carrying *AtTOR*. The independent nature of T-DNA integration into the genome of transgenic plants was identified by the different restriction fragments binding to the probe. Of the 12 samples analyzed, single insertions were found in 7 plants, while one plant carried two insertions (Fig. 2c). Few transgenic plants did not show any band, probably because of improper digestion or poor genomic DNA quality. The high expression plants, TR2.24 and TR15.10 selected for molecular investigations had a single copy of T-DNA integration in their genome.

### Semi-Q and qRT-PCR to separate *AtTOR* lines based on kinase expression

**Figure 3:**
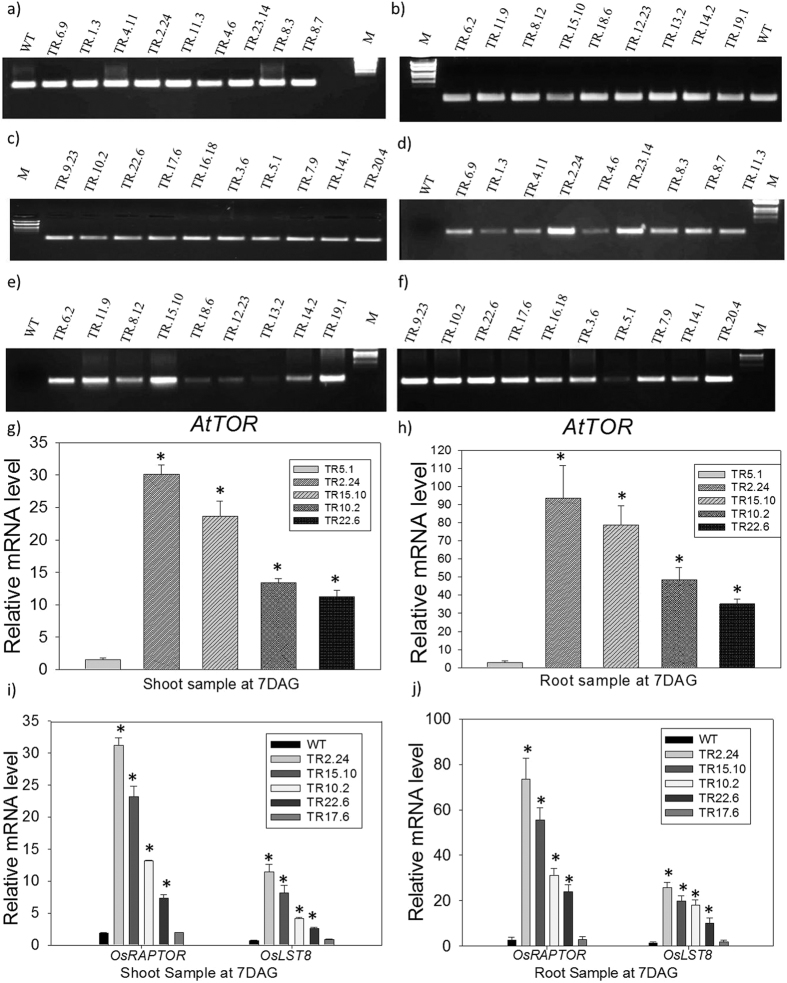
Semi-Quantitative and qRT-PCR analysis of *AtTOR* transgenic plants in T_2_ generation and expression analysis of TORC1 components. Rice actin (*Act1*) was used as an internal reference gene (**a–c**). *AtTOR* specific kinase was used to assess the transcript levels that amplifies only in *AtTOR* transgenic plants but not in WT rice (**d–f**). Based on the band intensity on the gel, lines TR5.1, TR18.6, TR12.23, TR1.3, TR4.6 and TR13.2 were considered as low expression lines. Lines TR6.9, TR4.11, TR8.3, TR8.7, TR11.3, TR11.9, TR10.2, TR6.2 TR19.1, TR9.23, TR22.6, TR16.8, TR7.9, TR14.1, and TR20.4 were considered as medium expression lines. Lines TR2.24, TR2.12, and TR15.10 were categorized as high expression lines. Line TR5.1 was used to normalize the expression pattern in (**g,h**) qRT-PCR. (**i,j**) The expression patterns of *OsRaptor* and *OsLst8* were studied in one low, two medium and two high expression lines. The relative expression was considered statistically significant at *P* value < 0.05 (represented with asterisks) based on one-way ANOVA in all the analyzed genes.

Based on the band intensity of *AtTOR* kinase domain specific amplification observed through semi-Q PCR, the *AtTOR* rice T_2_ generation transgenic plants were separated into low, medium and high expression lines. Rice *actin* was used to normalize the expression patterns (Fig. 3a–c). Based on the band intensity, rice lines were categorized into low, medium and high expression using Arabidopsis specific kinase (Fig. 3d–f). The transcript levels of the same were further determined by qRT-PCR using the line with low-band intensity to normalize the expression pattern of other lines. The lines with transcript levels up to 15-fold were considered as medium while more than 15-fold were considered as high expression lines. Two lines, TR-2.24 and TR-15.10, were identified as high expression lines, having transcript levels up to 30-fold in shoots and up to 80-fold in roots. Transgenic lines, TR-9.23, 22.6, 23.14, 10.2, 8.3, 6.2, 19.1 were categorized as medium with transcript levels up to 15-fold (Fig. 3g,h).

### Transcript analysis of OsTORC1 components in transgenic plants

Since OsRaptor and OsLst8 interact with HEAT repeats and the kinase domain of TOR protein, respectively, we analyzed the expression pattern of these two genes in root and shoot tissues of one low, two medium and two high expression lines. In the high expression lines, TR2.24 and 15.10, the transcript level of *OsRaptor* was more than 30-fold in shoots and up to 70-fold in roots whereas the transcript level of *OsLST8* in these lines was up to 12-fold in shoots and 25-fold in roots (Fig. 3i). In shoots, of medium expression lines, TR-22.6, and TR-10.2, the transcript level of *OsRaptor* and *OsLst8* ranged between 8 to 12 and 30 to 35-fold, respectively whereas the transcript levels of *OsRaptor* and *OsLst8* upregulated up to 20 and 25-fold, respectively in roots (Fig. 3j). The transcripts of these two genes in low expression line, TR5.1 were same as in WT. The expression levels of *OsRaptor* and *OsLst8* transcripts were in accordance with the lines selected based on the expression levels of *AtTOR* indicating that the *AtTOR* gene directly regulates the *Raptor* and *Lst8*.

### Phenotypic analysis of *AtTOR* transgenic plants

**Figure 4:**
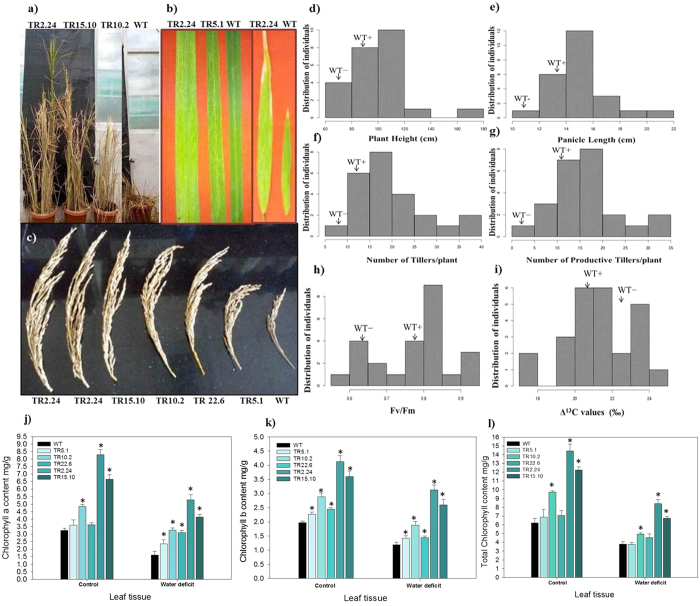
Phenotypic characterization, Quantum efficiency, ∆^13^C analysis and Chlorophyll estimation in 35 S: At*TOR* transgenic rice plants in T_2_ generation. (**a**) Phenotypic observations of high expression lines, TR2.24, TR.15.10; medium expression line, TR10.20 with respect to WT grown under limited water conditions. (**b**) Variation in size of boot leaf and (**c**) Panicle length. Graphical representation of (**d**) plant height, (**e**) panicle length, (**f**) number of tillers and the (**g**) number of productive tillers observed under limited water conditions. (**h**) Quantum efficiency of PSII in transgenic plants was measured under water-limited conditions. High expression transgenic lines, TR2.24 and TR15.10, had the highest quantum yield of >0.90 (**i**) Transgenic plants TR2.24, and TR 15.10 had low Δ^13^C values of 17.02‰ and 17.4‰, respectively under limited water conditions indicating their high WUE while WT grown under similar circumstances had a Δ^13^C value of 21.95‰. ∆^13^C was analyzed using IRMS. (**j**) Chlorophyll-a, (**k**) Chlorophyll-b content, and l) total chlorophyll content in low (TR5.1), medium (TR10.2, TR22.6) and high (TR2.24, TR15.10) expression lines were measured before and after growth of plants under limited water conditions. Mean values of chlorophyll data with ± SE represented with asterisks were considered statistically significant at *P* < 0.05.

We observed various growth and yield related parameters in low, medium and high expression lines such as the number of tillers, panicles, the length of panicles and total seed yield. All the phenotypic and physiological measurements of the transgenic plants were recorded after their growth under limited water conditions only and the readings were compared with two types of WT, one grown with adequate water and the other grown with limited water. The medium and high expression lines exhibited higher yield compared with the low expression lines and WT. After 45 d of continuous growth under limited water supply, WT started to undergo wilting, while the two high expression transgenic lines (TR2.24 and TR15.10), continued to stay green till plant maturity and seed harvest. These plants had increased tillering, plant height, panicle length and number of productive tillers under limited water conditions with respect to WT. The high expression lines, TR2.24, and TR15.10 reached a height of 166.2 cm and 138.6 cm, respectively (Fig. 4a), while medium expression lines, TR10.2, TR22.6, TR9.23, TR8.3, and TR23.14 had 116.7 cm, 113.6 cm, 111.5 cm and 108.4 cm 102.6 cm plant height respectively. The plant height in the low expression lines TR12.23, TR13.2, TR18.6, TR21.1, was 82.5 cm, 80.7 cm, 78.7 cm and 72.9 cm, respectively, compared with WT with a height of 68.2 cm.

The plant height of low expression line, TR5.1 was 66.8 cm which was almost equal to the WT. The high expression lines also had increased leaf area (Fig. 4b) and the panicle length in the range of 20.4 cm and 19.2 cm, respectively (Fig. 4c), whereas medium expression lines such as, TR10.2, TR22.6, TR9.23 and TR23.14 ranged from 14 cm to 17 cm and the low expression lines TR1.3, TR4.6, TR5.1, TR12.23, TR13.2, TR18.6 and TR21.1 ranged from 11 cm to 13 cm.

The number of tillers per plant in high expression lines, TR2.24, and TR15.10 was as high as 35, medium expression lines TR6.2, TR8.3, TR9.23 TR22.6 and TR10.2 had 20 to 30 tillers under limited water conditions while WT had 6–7 tillers. The number of productive tillers or panicles is a major factor that determines the overall yield of a plant. The high expression lines, TR2.24 and TR15.10 carried more than 30 productive tillers or panicles out of total 35 tillers while medium expression lines had 13 to 25 productive tillers. The yield and productivity in the two high expression lines of rice were more than that of WT grown with adequate water, suggesting that overexpression of TOR helps the plant in combating water deficiency and also plays a major role in maintaining or improving plant development and yield. The mean values of plant height (Fig. 4d), panicle length (Fig. 4e), the number of tillers (Fig. 4f), panicles (Fig. 4g) was plotted as histograms and these growth related parameters in different lines were detailed in Table 1.

### Photosynthetic efficiency of PSII in transgenic plants

The quantum yield of WT grown with adequate and limited water measured through MINI-PAM was in the range of 0.75–0.80 and 0.65, respectively while the quantum yield of the *AtTOR* rice transgenic plants grown under limited water conditions ranged from 0.65 in low expression lines to as high as 0.90 in high expression lines (Fig. 4h). Two transgenic lines, TR2.24, and TR15.10 had the highest quantum yield of 0.948 and 0.927, respectively while medium and low expressions lines ranged from 0.7 to 0.8 and 0.6 to 0.7, respectively indicating that *AtTOR* high expression lines were more photosynthetically active compared with WT under water-limited conditions.

### Carbon isotope (∆^13^C) analysis

The carbon isotope ratio 12C/13C (∆^13^C), is an important parameter to estimate the WUE of a plant, which is the amount of biomass produced per unit water transpired by the crop^36^. Low ∆^13^C values represent higher WUE^27^,^28^. In naturally occurring genotypes, high WUE is always associated with reduced biomass and yield, while in our study, we attempted to recombine the high yielding parameters under limited water availability. The high expression lines with higher biomass and yield had low ∆^13^C compared with WT grown under limited water conditions, indicating significantly high WUE (Fig. 4i). Lines, TR2.24 and TR 15.10 had lowest ∆^13^C value of 17.04‰ and 17.419‰, respectively as against the WT with 21.95‰ and 23.914‰ grown under adequate and limited water conditions, respectively. The medium expression lines showed considerable variation in ∆^13^C ranging from 19.26‰ to 20.728‰. The low expression *AtTOR* line, TR5.1 had highest ∆^13^C value, 24.295‰, which is almost at par with the WT. The photosynthetic quantum yield and Δ^13^C values measured in low, medium and high expression lines were presented in Table 1.

### Chlorophyll content in transgenic lines

The content of Chl-a in high expression lines, TR2.24 and TR 15.10 grown with sufficient water was observed up to 8 mg/g and 6 mg/g, respectively and this level was reduced after three weeks of water limiting conditions to 5 mg/g and 4 mg/g of leaf tissue (Fig. 4j). The medium expression lines, TR10.2 and TR22.6 had Chl-a content of 4.8 mg/g and 3.6 mg/g before and 3.2 mg/g and 3 mg/g after growth under water-limited conditions, respectively. The Chl-b content in high and medium expression lines was more than 3 mg/g and 2.5 mg/g before water stress while it was reduced to 2 mg/g and 1.5 mg/g, respectively (Fig. 4k) after growth under water deficit conditions. The total chlorophyll content in high expression lines was also as high as 14 mg/g and reduced to 8 mg/g, whereas the total chlorophyll content in medium expression lines, was more than 7 mg/g and up to 5 mg/g before and after growth under water-limited conditions (Fig. 4l). The reduction in Chl content under water stress conditions is a commonly observed phenomenon^37^, but the medium and high expression lines yet maintained sufficiently high chlorophyll content even after their growth under limited water conditions. The percent degradation of Chl-a, and -b was more in WT (50%) grown under limiter water availability followed by low-expression lines (40–45%). The percent degradation in high expression lines were as low as 10–15% (Supplementary Fig. 2a,b).

### Glucose-mediated activation of TOR

Glucose is an important plant regulatory molecule and linked to TOR activation in Arabidopsis root meristem^12^,^13^. Reports have suggested that glucose increases the TOR activity by regulating the binding of v-ATPase to Rag-GTPases suggesting a link between regulation of TOR by sugars and amino acids^38^. High expression lines, TR2.24, and TR15.10 showed a maximum number of lateral roots 5 DAG on glucose medium compared with the seedlings grown without glucose (Supplementary Fig. 3a,b). These two lines grown without glucose showed significantly higher lateral roots with respect to WT grown with glucose. The transcript levels of the kinase domain of TOR gene in these two lines increased up to 40-fold in shoots and up to 90-fold in roots (Supplementary Fig. 3c,d). The TOR levels increased to more than 10-fold in both shoot and roots of transgenic lines and WT grown with glucose compared with transgenic plants and WT grown without glucose, suggesting that like amino acids, glucose also activates the transcription of TOR in rice.

### ABA insensitivity of high *AtTOR* expression transgenic lines of rice

ABA is a stress hormone, which has an important role in the plants response under stress conditions^39^. Under water stress conditions, stomatal closure is an immediate response, which is controlled by ABA^40^. The direct role of TOR gene in ABA signaling under stress conditions is not yet studied, but the interaction of SnRKs and TOR has been reported^41^. The SnRKs (Sucrose-non-fermenting-1-related protein kinase-1) are metabolic sensors for energy deficiency signals^42^. The SnRK2 and SnRK3 are unique to plants and are also involved in plant response to several stresses^43^. Overexpression of SnRK2 participates in ABA signaling, response to cold and drought tolerance^44^,^45^ and it also transcriptionally upregulates several transcription factors and other stress responsive genes^45^. The seed germination assay of low, medium and high expression lines and WT on ABA (50 μM) showed that high and medium expression lines germinated and continued to grow normally indicating that they are insensitive to ABA treatment, whereas the low expression line along with WT was found to be sensitive to ABA. The germination rate of WT and transgenic lines grew on MS without ABA was 95% (Supplementary Fig. 3e). Whereas, the germination rate of high (TR2.24) and medium (TR10.2) expression lines on ABA was more than 80% measured after 3 and 5 DAG while the germination of WT and low expression line, TR5.1 was less than 40% on ABA (Supplementary Fig. 3f).

### Transcriptional regulation of stress-specific genes by *AtTOR* in high expression lines

To determine the role of *AtTOR* in the regulation of stress tolerance, we studied the expression pattern of genes specific or common to two or more abiotic conditions in two high expression lines of *AtTOR* under both treated and untreated conditions. The expression pattern of these stress-specific genes in untreated seedlings of high expression lines was normalized with untreated seedlings of WT, whereas treated seedlings of high expression lines were normalized with treated WT seedlings so that the level of expression of stress-specific genes in transgenic lines was due to the regulation by TOR gene.

One week old seedlings of two high expression lines were subjected to three abiotic treatments; ABA, PEG and NaCl and the transcript levels of 15 stress-specific genes have been characterized in the root and shoot tissues using qRT-PCR. We found that genes involved in osmotic protection such as *OsDHODH1, OsNADPH1, OsSKIP1a, OsALDH2a, OsAOX1a, OsTPP1, OsLEA3-1, OsGL1-2*, post-transcriptional modifications such as *OsPP2C* and *OsSIK1* and drought responsive transcription factors such as *OsNAC1, OsNAC2, OsWRKY72, OsDREB2B, OsbZIP23* were highly upregulated in high expression lines, TR2.24 and TR15.10 in both treated and untreated conditions. The level of expression was almost similar in shoot and root tissues of both the lines. These results suggest the possible role of *AtTOR* in transcriptional regulation of stress-responsive genes (Supplementary Fig. 4).

**Figure 5:**
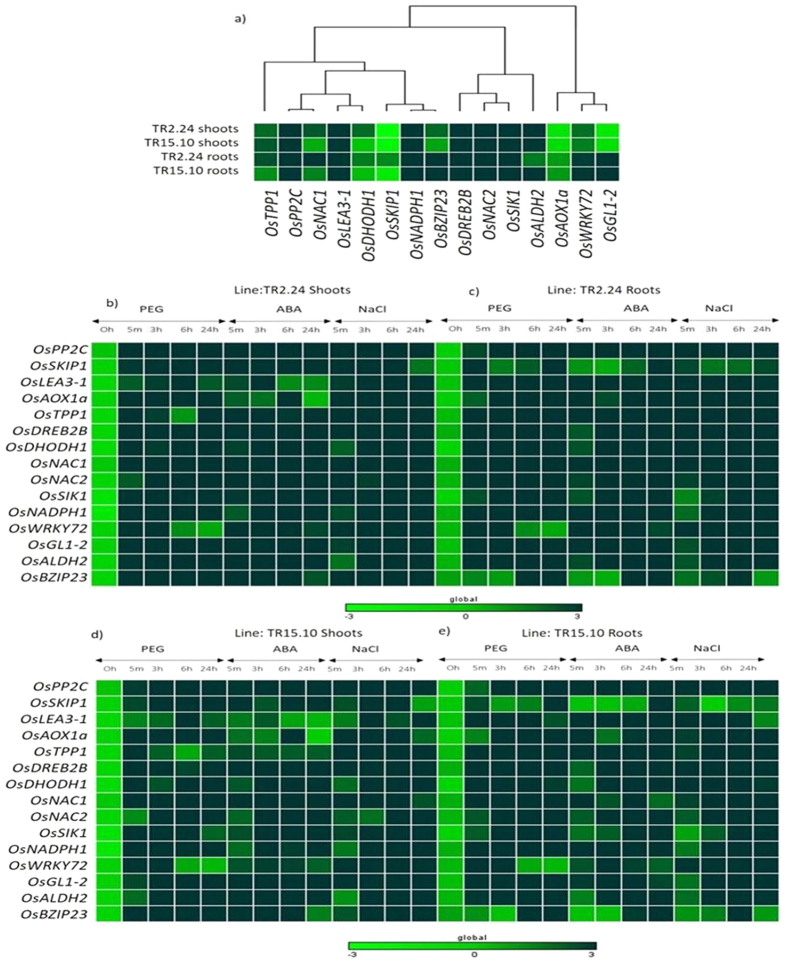
Heat map depiction of stress-specific genes in high expression lines of transgenic rice carrying *AtTOR.* The heat map was generated using GENE-E program to represent the transcriptional regulation of 15 stress-specific genes in (**a**) untreated and ABA, PEG, NaCl-treated shoots and roots of high expression lines (**b,c**) TR2.24 and (**d,e**) TR15.10.

Most of the genes analyzed in the *AtTOR* transgenic plants also showed some constitutive upregulation in untreated conditions except for the genes *OsGL-2, OsSKIP-1a, OsAox-1a* in the shoot tissues, while roots showed upregulation of these genes. However, the expression of all the genes became many fold upregulated in both shoot and root tissues in all the treatments, and this upregulation has been observed within 5 min treatment after treatment. The levels of upregulation have been represented in the form of Heat Maps (Fig. 5).

The transcript level of *OsDHODH1* in shoots of *AtTOR* lines TR2.24 and TR15.10 was up-regulated above 50-fold after 24 h of PEG treatment, while under ABA and NaCl treatments, the *OsDHODH1* transcript level was upregulated to 80 and 60-fold respectively after 3 h after the onset of the treatment. The transcript levels of *OsNADPH1* in shoots and roots of high expression lines was significantly upregulated up to 90- and 110-fold respectively after 3 h of NaCl treatment, while after 24 h of ABA and PEG treatments in shoots and after 6 h in roots, the mRNA level was more than 50 and 90–fold respectively. The *OsSKIP1a, OsAOX1a, OsDHODH1,* and *OsALDH2* were upregulated more than 50-fold in shoots of high expression lines after 24 h of PEG treatment, whereas *OsPP2C, OsLEA3-1* showed increased transcript level up to 10-fold and *OsNAC2* was upregulated up to 100-fold upregulation after 6 h of PEG treatment in shoots. The transcription factors *OsDREB2B* and *OsbZIP23* had 90 and 100-fold, respectively after 3 h of PEG treatment in shoots while other such as *OsNAC1* and *OsWRKY72* were upregulated as early as 5 min after immediate exposure to dehydration stress in shoots. The other genes such as *OsLEA3-1* and *OsNAC2* were upregulated up to 50-fold after 6 h of PEG treatment in roots. PEG-induced the expression of *OsPP2C* up to 80, *OsNADPH1* up to 60, *OsNAC1* up to 87 after 3 h of stress treatments. Under ABA treatment, *OsPP2C, OsDREB2B,* and *OsNADPH1* were significantly upregulated up to 70-fold after 24 h in the shoot, while the transcript levels of *OsALDH2 w*ere upregulated 20-fold and *OsNAC1, OsNAC2, OsSIK1, OsGL1-2,* and *OsbZIP23* were more than 25-fold after 6 h of treatment in shoots. Similarly, the *OsLEA3-1* and *OsWRKY72* was upregulated after 3 h of ABA treatment in shoots up to 8-fold in shoots. The root tissues of high expression lines, under ABA treatment, showed a transcript level of *OsPP2C* up to 96-fold immediately after 5 min; *OsDREB2B* and *OsbZIP23* up to 60-fold and 47-fold after 24 h of treatment respectively, whereas *OsNAC1*and *OsSKIP1a* expression level was increased up to 9-fold and 33-fold after 24 h of ABA treatment. In the roots of two high expression lines, genes such as *OsALDH2, OsGL1-2* and *OsNAC2* were highly regulated 6 h post treatment to ABA. The transcript level of *OsNADPH1* in shoots and roots of both transgenic lines were significantly upregulated above 70-fold at 3 h after 300 mM NaCl treatment.

### Promoter and protein phylogenetic analysis of TOR

The upstream 1500 bp regions of *OsTOR* and *AtTOR* were found to have multiple *cis-*regulatory elements that respond to plant signaling molecules and stress treatments. Sequence alignment of the two putative promoter elements of rice and Arabidopsis showed 45% nucleotide similarity, indicating the presence of similar motifs. Elements that respond to hormones such as ABA, (ABRE), salicylic acid (TCA element), auxin (AuxRR) and methyl jasmonate (CGTCA and TGACG motifs) are distributed widely within the putative promoter of *OsTOR*. Also, heat stress responsive element (HSE) and two repeats of MYB binding sites, involved in drought-induced response are also present (Supplementary Fig. 5a,b).

The phylogenetic analysis of protein sequences derived from a diverse range of eukaryotes showed that TOR gene evolved from a possible common ancestor. All the three subspecies of rice were present on the same clade showing high similarity within the family. The *TuTOR* showed similarity with *OsTOR*, whereas *ZmTOR* and *SbTOR* exhibited high similarity with each other compared with *OsTOR*. The other members such as *VrTOR* and *AdTOR* belonging to Fabaceae, *AtTOR* and *BnTOR* belonging to Brassicaceae showed high similarity to the members of the same family (Supplementary Fig. 5c). The phylogenetic tree had been generated using MEGA7 program by the text neighbor joining tree option with a boot strap value of 1000.

## Discussion

TOR is a multifunctional protein, with roles in the regulation of diverse signaling pathways. The *AtTOR* overexpression and RNAi-mediated silencing directly influenced Arabidopsis growth, seed yield, osmotic stress tolerance, ABA and sugar sensitivity as well as polysome accumulation^2^,^46^. The overexpression of the *AtTOR* resulted in increased biomass, shoot growth, leaf size, increased inflorescence size, seed production and resistance to metabolic and osmotic stresses^1^,^2^ while *AtTOR* disruption showed retarded shoot growth and root development after germination in Arabidopsis^12^,^13^,^14^,^47^.

TOR protein positively regulates biosynthetic pathways such as cell wall modification, cell cycle, carbon and nitrogen utilization, photosynthesis and nutrient transport but negatively regulates catabolic processes such as autophagy and senescence^47^. Glucose-activated TOR signaling networks regulate a large number of conserved chromatin modulators, signaling regulators, transcription factors and growth and stress-related proteins in eukaryotes and thereby helps in mitigating environmental stresses^2^,^14^.

Since the full-length sequence of *OsTOR* was not available at the initiation of this research activity, we have used the functionally characterized and available Arabidopsis *AtTOR* sequence and gene constructs to explore effects of ectopic expression of this heterologous but highly conserved plant representative *TOR* sequence in relation to WUE in *indica* rice in the present study.

In the present study, the overexpression of *AtTOR* resulted in increased biomass and higher yield potential under limited water conditions. The plant biomass and yield have been in accordance with the level of *AtTOR* expression. An increase in *AtTOR* expression increased the biomass with high tillering, panicle length, panicle number and leaf area under limited water conditions, whereas, the low expression lines exhibited phenotypes similar to the WT plants, indicating that the TOR protein works in a dose-dependent manner, and its level of expression is directly linked to the phenotypes. The high *AtTOR* expression lines exhibited sustained or improved growth and productivity under limited water conditions compared with WT, which started wilting after three weeks of water deficit conditions, suggesting that *AtTOR* has the potential to improved yield under limited water conditions.

Previous reports showed that yeast TORC1 activity inhibited in response to carbon, nitrogen, phosphate deficiency, high salinity, high temperatures and oxidative stresses^48^. This downregulation of TOR gene under stresses is probably to slow down the energy consumption or nutrient uptake under stress conditions to extend the survival of plants. In plants, TOR is negatively regulated by stress, but the high transcript levels of TOR in transgenic overexpression lines could likely maintain a balance in the normal growth even under stress conditions. Also, TOR mitigates the effects of stress by positively modulating the expression of multiple stress-specific genes, which act concurrently in plants defense against various stresses.

The knowledge on the mechanism, signaling pathway and genes involved in TOR signaling in plants remain to be unravelled. The *AtTOR* overexpression lines exhibited increased primary roots to relieve the stress caused by excess nitrogen^2^. The ectopic expression of TOR regulatory component *AtRaptor1* and TOR substrate *AtS6K1,* which is sensitive to osmotic stress in tobacco increased osmotic stress tolerance by relieving *AtS6K1* sensitivity^49^. Our observations show that the transcript levels *OsTOR* regulatory elements; *OsRaptor* and *OsLST8* increased with an increase in the level of *AtTOR* transcripts, likely indicating the function of TOR in regulation of its own machinery components. All plants have been reported to be insensitive to Rapamycin^12^,^14^,^50^, but maize seeds were found to be sensitive to rapamycin^18^. The WT rice plants were germinated on four different concentrations of Rapamycin from 20 μg/mL to 250 μg/mL, but the germination and growth were not affected at even higher concentrations, indicating the insensitivity of *OsTOR* to Rapamycin. This insensitivity was further confirmed by phosphorylation assay of S6K1, which is an immediate and direct downstream target of TOR in Rapamycin (200 and 250 μg/mL) treated WT rice seedlings. This Rapamycin insensitivity in rice might be due to improper Rapamycin- FKBP12 binding or structural changes in *Os FKPB12*. The overexpression of *TOR* protein or kinase domain alone in Arabidopsis and rapeseed resulted in increased WUE^1^,^51^,^52^. In our study, the high expression lines, TR2.24 and TR15.10 exhibited ∆^13^C values as low as 17.02‰ and 17.41‰ under water deficit conditions compared with WT grown under adequate (21.95‰) and limited water conditions (23.914‰). The low expression lines, TR.5.1 had highest ∆^13^C value, 24.26‰ which is at par with WT. These observations confirm the critical role of TOR protein in enhancing WUE, which is also directly linked to its expression levels. *AtTOR* high expression lines also showed a high quantum yield of Photosystem II up to 94% under water deficit conditions compared to WT, which is around 68% while WT grown with adequate water supply showed 75–80% quantum yield of photosystem II. Drought stress induces considerable damage to the photosynthetic pigments, thus reducing the photosynthetic efficiency and chlorophyll content of plants^37^. The chlorophyll content in *AtTOR* transgenic lines along with WT was decreased up to 50% after three weeks of growth under water deficit conditions. However, the high and medium lines continued to maintain sufficiently elevated levels of both Chl-a, and Chl-b indicating the minimal damage to chlorophyll and hence, chloroplasts. These observations showed that high expression level of *AtTOR* increased the photosynthetic activity even under water stress conditions. Expression studies of stress-specific genes in high *AtTOR* expression seedlings subjected to various stress treatments suggested that TOR positively regulates the expression of genes involved in plant response to stress.

Branched-chain amino acids such as leucine, isoleucine, and valine or leucine are reported as important regulators of translation initiation by activating TOR signaling pathways in mammals^53^. Amino acids such as serine, threonine, valine, leucine, isoleucine, arginine, alanine, glutamine, and proline up-regulated the *OsTOR* expression, whereas amino acids such as tryptophan, histidine, cysteine, lysine downregulated the *OsTOR* expression in roots and shoot.

The activation of TOR by glucose is necessary for root growth and activation of quiescent root meristem^12^. Germination of *AtTOR* rice transgenic lines and WT on glucose had increased the lateral root growth and the transcript levels of the *TOR* gene. The medium (TR10.2) and high (TR2.24) expression lines were found to be insensitive to phytohormone, ABA compared with WT and low expression line (TR5.1) indicating that the high transcript levels of TOR might reduce the ABA sensitivity.

We studied the transcriptional regulation of 15 different stress-specific genes involved in osmotic protection and plants defense. The high and significant upregulation of all the 15 genes investigated in two high expression lines under both untreated and stress-treated conditions indicate that TOR alleviates stress and induce plants tolerance to stress by concurrently upregulating multiple stress-specific genes. In conclusion, our observations on transgenic rice expressing *AtTOR* showed that its high expression exhibited beneficial effects on the plant regarding high WUE, which was associated with the overall increase in the biomass and yield of the plant. Manipulation of *TOR* could have significant consequences for stress management and plant performance.

## Additional Information

**How to cite this article:** Bakshi, A. *et al*. Ectopic expression of *Arabidopsis Target of Rapamycin (AtTOR*) improves water-use-efficiency and yield potential in rice. *Sci. Rep.* **7**, 42835; doi: 10.1038/srep42835 (2017).

**Publisher's note:** Springer Nature remains neutral with regard to jurisdictional claims in published maps and institutional affiliations.

## Supplementary Material

Supplementary Information

## Figures and Tables

**Table 1:** Phenotypic characterization, Quantum yield, and Δ^13^C values of low, medium and high *AtTOR* expression rice transgenic lines with respect to wild type.

S No.	Line Name	Plant height (cm)	Panicle length (cm)	No. of tillers	No. of productive tillers	Quantum Yield ± SE	Δ^13^C values (‰)
1	TR.1.3*	89.1 ± 1.453	13.6 ± 0.9262	15.1 ± 0.6083	11.1 ± 0.5196	0.732 ± 0.042557	22.186
2	TR.2.24***	166.2 ± 3.1798	20.4 ± 0.636	35.3 ± 0.8192	33.4 ± 0.6083	0.948 ± 0.080898	17.022
3	TR.3.6**	96.2 ± 0.7265	14.1 ± 0.5487	19.2 ± 1.105	15.6 ± 0.3383	0.755 ± 0.029627	21.466
4	TR.4.6*	85.6 ± 0.318	13.7 ± 1.6384	12.5 ± 0.809	10.3 ± 0.6351	0.697 ± 0.068394	22.874
5	TR.5.1*	66.8 ± 0.6227	14 ± 0.5774	12.5 ± 1.105	9.2 ± 0.6121	0.559 ± 0.044096	24.295
6	TR.6.2**	103 ± 2.7285	15.5 ± 1.1547	22.8 ± 1.618	19.3 ± 0.3383	0.811 ± 0.029059	20.655
7	TR.7.9**	99.8 ± 0.8192	14.9 ± 1.3317	19.2 ± 1.3618	16.4 ± 0.9207	0.802 ± 0.025207	21.34
8	TR.8.3**	104.1 ± 0.8413	15.7 ± 0.5783	23.3 ± 0.7881	18.6 ± 0.5508	0.828 ± 0.054874	20.151
9	TR.9.23**	111.5 ± 3.493	16.4 ± 1.3618	28 ± 0.7	23.9 ± 0.6674	0.848 ± 0.029059	19.362
10	TR.10.2**	116.7 ± 1.6384	16.9 ± 0.9539	32 ± 1.0477	29.7 ± 0.8452	0.908 ± 0.029627	19.095
11	TR.11.9**	103.8 ± 2.4552	15.8 ± 0.809	22 ± 0.7688	17 ± 0.835	0.807 ± 0.054874	20.294
12	TR.12.23*	82.5 ± 1.1552	13.2 ± 0.5487	13 ± 0.4055	10.3 ± 0.8819	0.618 ± 0.060645	23.076
13	TR.13.2*	80.7 ± 1.8586	13.7 ± 1.4422	13.2 ± 1.0975	11.6 ± 0.4667	0.621 ± 0.041633	23.082
14	TR.14.1**	95.4 ± 1.4449	14.2 ± 0.9333	18.6 ± 0.5783	14.8 ± 0.6028	0.758 ± 0.057831	21.478
15	TR.15.10***	138.6 ± 1.7436	19.2 ± 0.5487	36.8 ± 1.0975	30.3 ± 0.6083	0.927 ± 0.075719	17.418
16	TR.16.18**	101.8 ± 2.0518	15.1 ± 1.0408	19.2 ± 0.4807	16.6 ± 0.2848	0.848 ± 0.058119	21.074
17	TR.17.6**	99.6 ± 1.3618	14.6 ± 0.5487	19.7 ± 2.0224	14.6 ± 0.3844	0.817 ± 0.046667	21.517
18	TR.18.6*	78.7 ± 1.0729	13.6 ± 1.3018	11.4 ± 1.105	9.3 ± 0.3844	0.650 ± 0.067412	23.4
19	TR.19.1**	102 ± 1.7776	15.4 ± 1.3618	22.8 ± 1.0975	16.6 ± 0.5508	0.797 ± 0.070946	20.728
20	TR.20.4**	100.9 ± 2.4556	15 ± 1.105	19.8 ± 0.6888	16.2 ± 0.6083	0.832 ± 0.041633	20.131
21	TR.21.1*	72.9 ± 0.5812	13.5 ± 0.318	11.6 ± 0.5508	9 ± 0.6429	0.638 ± 0.041633	23.77
22	TR.22.6**	113.6 ± 1.4449	16.8 ± 0.8718	28.2 ± 1.1269	23.6 ± 0.7172	0.873 ± 0.041633	19.26
23	TR.23.14**	102.6 ± 1.1136	15.9 ± 1.105	15.1 ± 0.6083	13 ± 0.3227	0.782 ± 0.020276	20.57
24	WT^+^	89.3 ± 1.054	14.3 ± 1.205	12.2 ± 0.224	10.5 ± 1.032	0.803 ± 0.057831	21.95
25	WT^¯^	68.2 ± 1.618	11.8 ± 0.441	8.5 ± 0.5774	4.6 ± 0.7219	0.632 ± 0.052831	23.914

Phenotypic observations included, the number of tillers, panicle length, productive tillers (seed yielding panicles) and plant height were recorded as the mean of 5 plants of the corresponding low, medium and expression line with respect to WT. Transgenic lines TR2.24, TR15.10, had highest seed yield under limited water conditions. Quantum yield of PSII was taken as the mean of three values, each measured at 15 d intervals after growth of plants under limited water conditions. Carbon isotope analysis (Δ^13^C) of low, medium and high expression transgenic lines after six weeks of withdrawing water along with WT. The high expression line, TR2.24 showed lowest Δ^13^C value. WT^+^; WT grown with adequate water conditions, WT^−^; WT grown under limited water (High expression lines indicated as ***, medium expression lines as ** and low expression lines as *).
