# Corrigendum: Ectopic expression of *Arabidopsis Target of Rapamycin (AtTOR*) improves water-use efficiency and yield potential in rice

**DOI:** 10.1038/srep46124

**Published:** 2017-04-10

**Authors:** Achala Bakshi, Mazahar Moin, M. Udaya Kumar, Aramati Bindu Madhava Reddy, Maozhi Ren, Raju Datla, E. A. Siddiq, P. B. Kirti

Scientific Reports 7: Article number: 4283510.1038/srep42835; published online: 02 23 2017; updated: 04 10 2017

This Article contains errors in the Affiliations of Maozhi Ren and Raju Datla. The correct affiliations for these authors are listed below:

Maozhi Ren

Biotechnology Research Institute, Chinese Academy of Agricultural Sciences, Beijing, P.R. China; School of Life Sciences, Chongqing University, Chongqing, P.R. China.

Raju Datla

Plant Biotechnology Institute, National Research Council of Canada, Saskatoon, Saskatchewan, S7N 0W9, Canada.

