# Supplementary material for: Ectopic expression of Arabidopsis Target of Rapamycin (AtTOR) improves water-use efficiency and yield potential in rice
Source: Sci Rep. 2017 Feb 23;7:42835. doi: 10.1038/srep42835 (PMC5322334; doi:10.1038/srep42835)
Supplement: Supplementary Information [file srep42835-s1.pdf]

Ectopic expression of *Arabidopsis Target of Rapamycin (AtTOR)* improves water-use efficiency and yield potential in rice

Achala Bakshi[1], Mazahar Moin[1], M. Udaya Kumar[2], Aramati Bindu Madhava Reddy[3], Maozhi Ren[4], Raju Datla[5], E.A Siddiq[6] and P B Kirti[1*]

[1]*Department of Plant Sciences, University of Hyderabad, Hyderabad-500046, India*

[2]*Department of Crop Physiology, University of Agricultural Sciences-GKVK, Hebbal, Bangalore, India*

[3]*Department of Animal Biology, University of Hyderabad, Hyderabad-500046, India*

[4]*Plant Biotechnology Institute, National Research Council of Canada, Saskatoon, Saskatchewan, Canada S7N 0W9*

[5]*Biotechnology research Institute, Chinese academy of Agricultural Sciences, Beijing, PR China School of Life Sciences, Chongqing University, Chongqing, PR China*

[6]*Institute of Agricultural Biotechnology, PJTS Agricultural University, Rajendranagar, Hyderabad-500030, India*

[*]*Author for Correspondence, E-Mail: pbkirti@uohyd.ac.in, Phone: +91-40-23134545*

Authors contact details:

Achala Bakshi: achalabakshi@gmail.com

Mazahar Moin: moinmazahar@gmail.com

Udaya Kumar: udayakumar_m@yahoo.com

A.Bindu Madahava reddy: binduaramati@gmail.com

Raju Datla: raju.datla@nrc-cnrc.gc.ca

Maozhi Ren: renmaozhi@cqu.edu.cn

E A Siddiq: easiddiq@gmail.com

Short running title: *AtTOR* enhances water-use-efficiency in rice

Supplementary Figure 1

Transcriptional regulation of *TOR* by different amino acids, germination of WT on MS supplemented with Rapamycin and TOR activity based on OsS6K phosphorylation

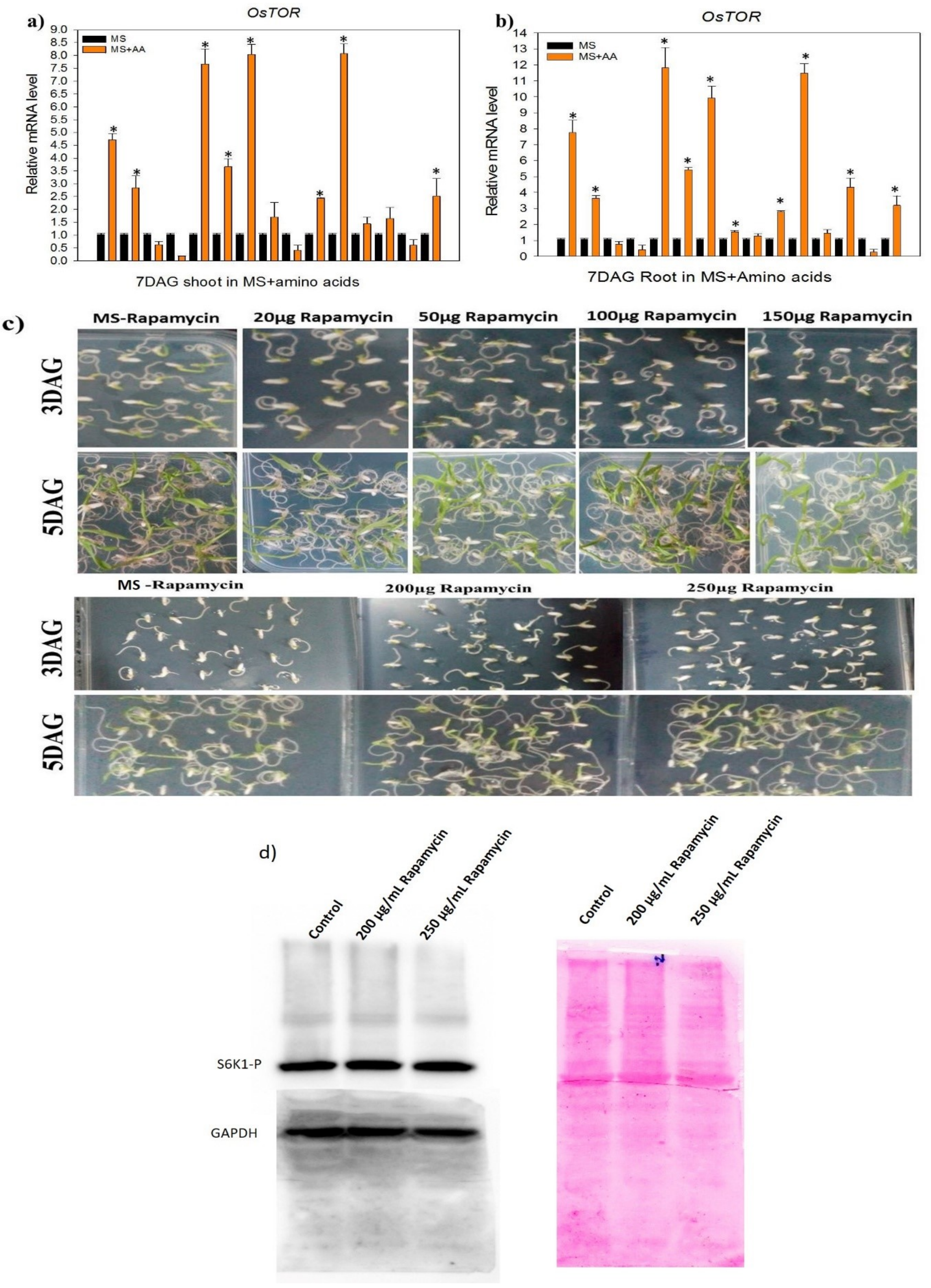

Fig. 1: Out of 15 amino acid treatments, *OsTOR* responded to all the treatments in (a) shoots and (b) root tissues of WT rice seedlings. Amino acids such as cysteine, tryptophan, histidine and lysine downregulated the expression of *OsTOR*, whereas other amino acids induced its upregulation. The fold-change of transcripts is a mean of three biological replicates. The relative expression was considered statistically significant at *P* value <0.05 (represented with asterisks) based on one-way ANOVA in all the analyzed genes. (c) Germination of WT seeds on MS and MS supplemented with six different concentrations; 20 µg/mL, 50 µg/mL, 100 µg/mL, 150 µg/mL, 200 µg/mL and 250 µg/mL of Rapamycin. The WT seed germination and growth was not affected at even 250 µg/mL concentration of Rapamycin. (d) The full-length Western blot analysis of Rapamycin treated (200 µg/mL & 250 µg/mL) and Untreated (control) seedlings using human anti-phospho-p70S6K (Thr(P)-389) and anti-GAPDH antibody.

Supplementary Figure 2

Chlorophyll a and b degradation percentage in low, high and medium *AtTOR* lines compared with WT after growth under limited water conditions.

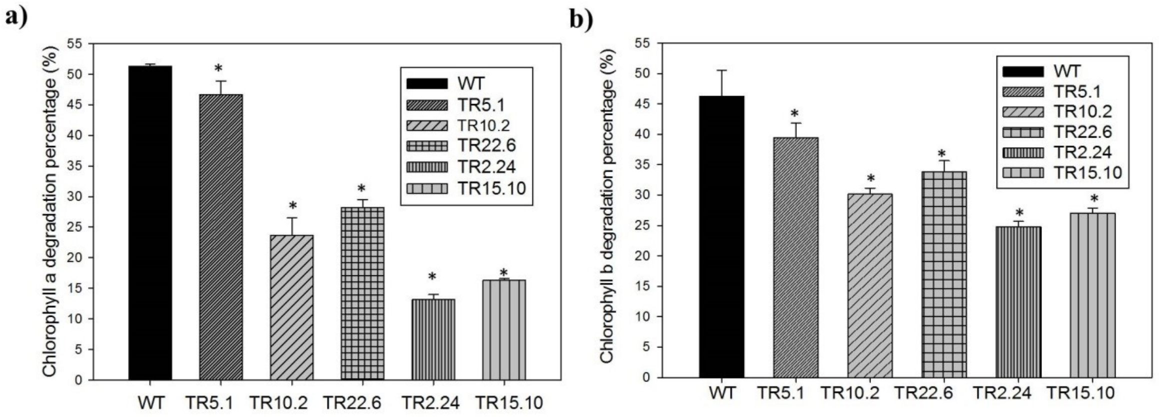

.

Fig. 2 (a) Percent degradation of chlorophyll a content in low (TR5.1), medium (TR10.2, TR22.6) and high (TR2.24, TR15.10) *AtTOR* lines compared to WT after water stress treatments. **(b)** Percent degradation of chlorophyll b content in low (TR5.1), medium (TR10.2, TR22.6) and high (TR2.24, TR15.10) *AtTOR* lines compared to WT after water stress treatments. The mean value of chlorophyll degradation percentage was considered statistically significant at $P<0.05$, represented with asterisks.

Supplementary Figure 3

Glucose mediated activation of TOR enhances lateral root growth and ABA insensitivity of *AtTOR* rice transgenic lines

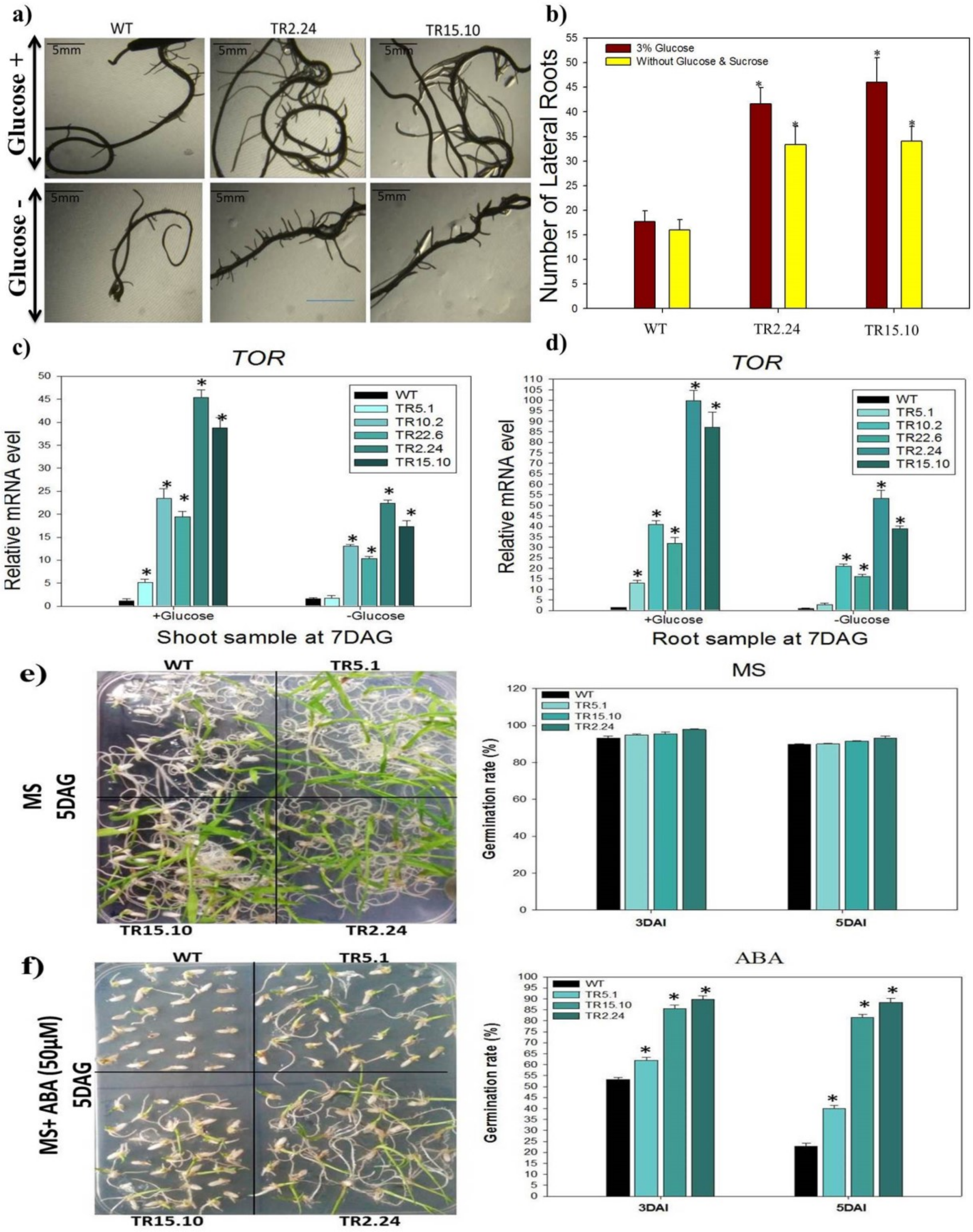

Fig. 3: (a) Initiation of lateral root growth in high *AtTOR* rice lines, TR2.24 and TR15.10 and WT on medium supplemented with 0.3% Glucose compared with WT. The high expression lines exhibited lateral root growth in medium without glucose similar to WT grown with glucose. (b) Number of lateral roots in high *AtTOR* rice lines, TR2.24 and TR15.10 and WT on MS medium supplemented with 0.3% Glucose compared to medium without glucose. The mean value of lateral roots of five seedlings were considered statistically significant at $P<0.05$, represented with asterisks. (c,d) The expression analysis of TOR gene in shoot and root tissues of low (TR5.1), medium (TR10.2, TR22.6) and high (TR2.24 and TR15.10) lines grown on MS with and without glucose. The relative expression was considered statistically significant at P value <0.05 (represented with asterisks) based on one-way ANOVA in all the analyzed genes. (e) The low (TR5.1), and high (TR2.24, TR15.10) *AtTOR* expression rice transgenic lines along with WT rice seeds were germinated initially on MS without ABA. The germination rate was scored and plotted as a bar diagram with ± standard deviation and represented with asterisks at $P<0.05$. (f) The low (TR5.1) and high (TR2.24, TR15.10) *AtTOR* expression rice transgenic lines along with WT rice seeds were allowed to germinate on ABA (50 µM), and the germination rate was scored and plotted as a bar with ±SD. The statistical significance was calculated using one-way ANOVA at $P<0.05$ and represented with asterisks.

Supplementary Figure 4

Transcriptional regulation of stress-specific genes by *AtTOR* in high expression lines

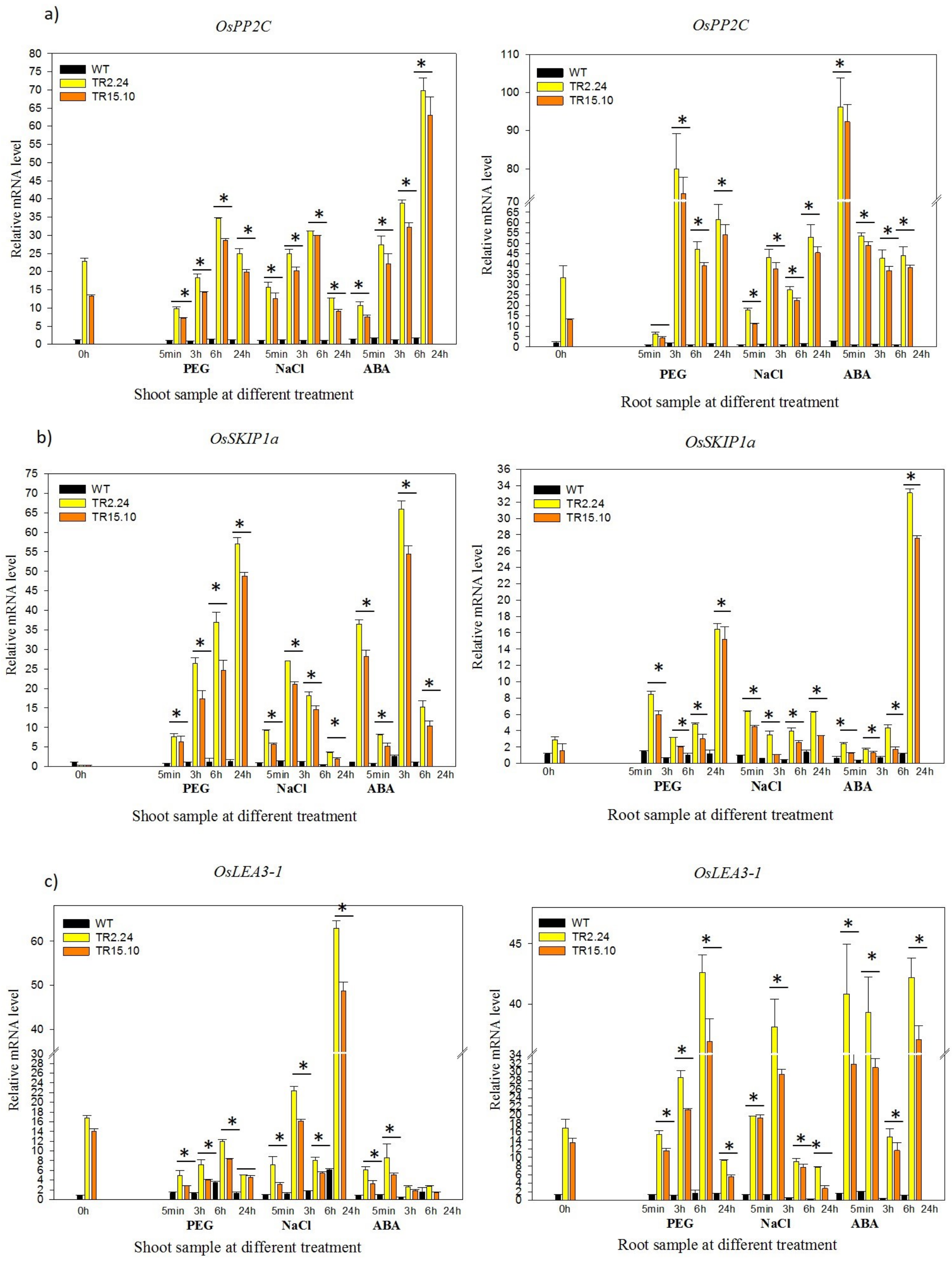

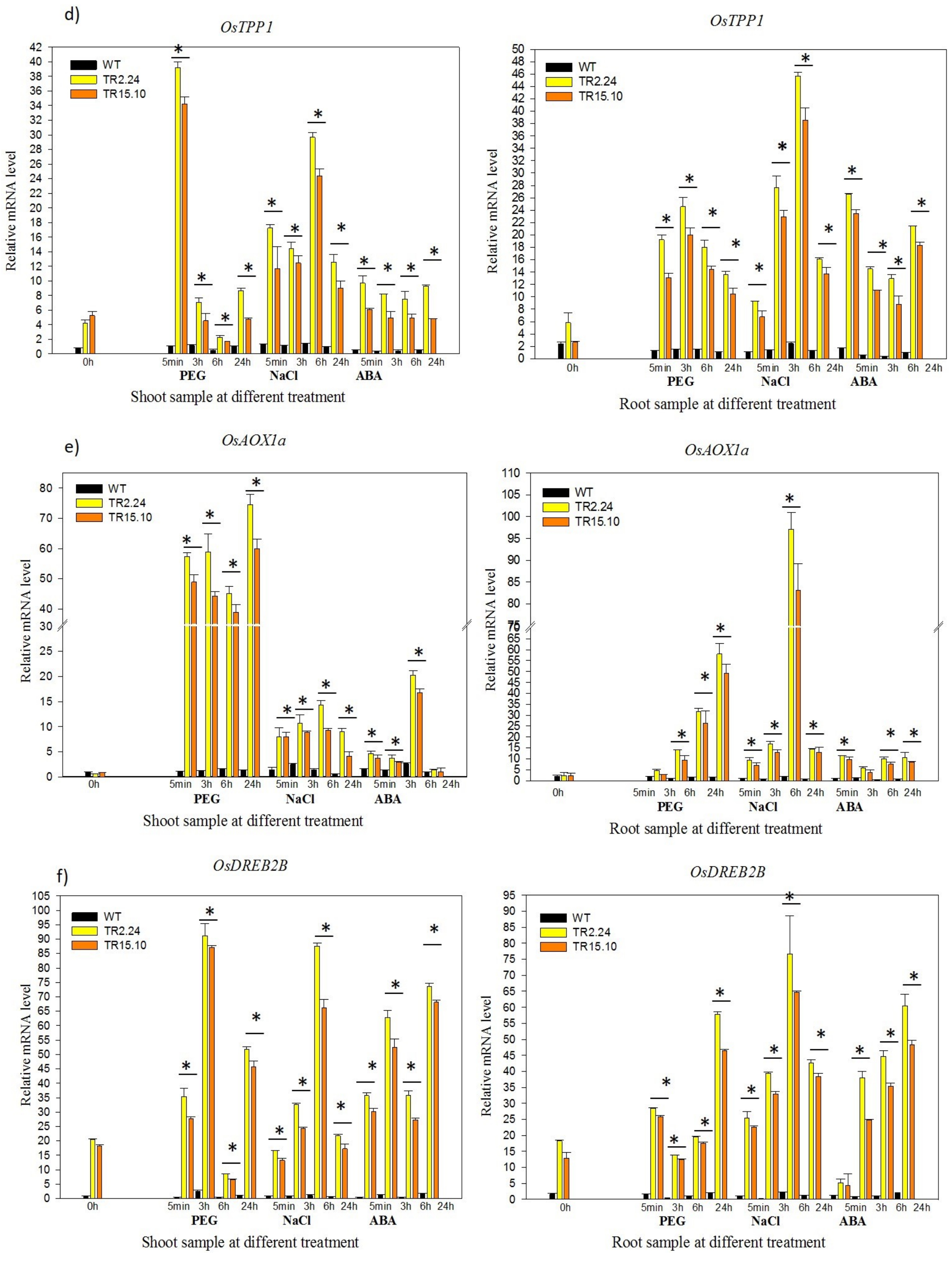
d)
OsTPP1
WT
TR2.24
TR15.10
Relative mRNA level
0h
5min 3h 6h 24h
PEG
NaCl
ABA
Shoot sample at different treatment
OsTPP1
Root sample at different treatment
e)
OsAOX1a
Shoot sample at different treatment
OsAOX1a
Root sample at different treatment
f)
OsDREB2B
Shoot sample at different treatment
OsDREB2B
Root sample at different treatment

g)

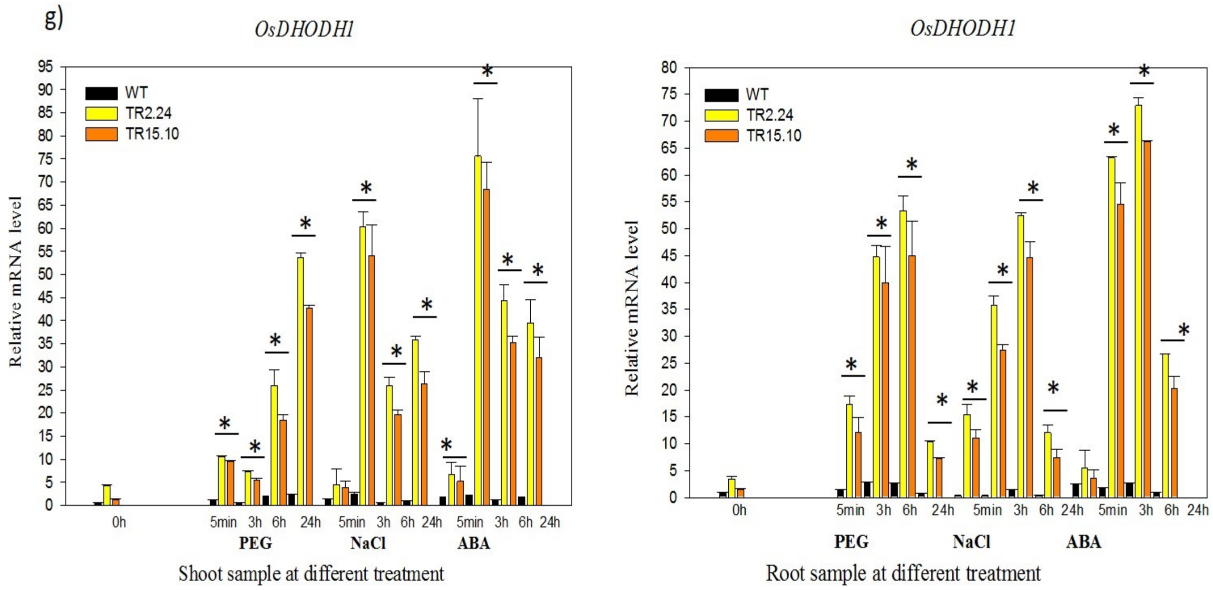

OsDHODH1
WT
TR2.24
TR15.10
Relative mRNA level
0h
5min 3h 6h 24h 5min 3h 6h 24h 5min 3h 6h 24h
PEG NaCl ABA
Shoot sample at different treatment
OsDHODH1
WT
TR2.24
TR15.10
Relative mRNA level
0h
5min 3h 6h 24h 5min 3h 6h 24h 5min 3h 6h 24h
PEG NaCl ABA
Root sample at different treatment

h)

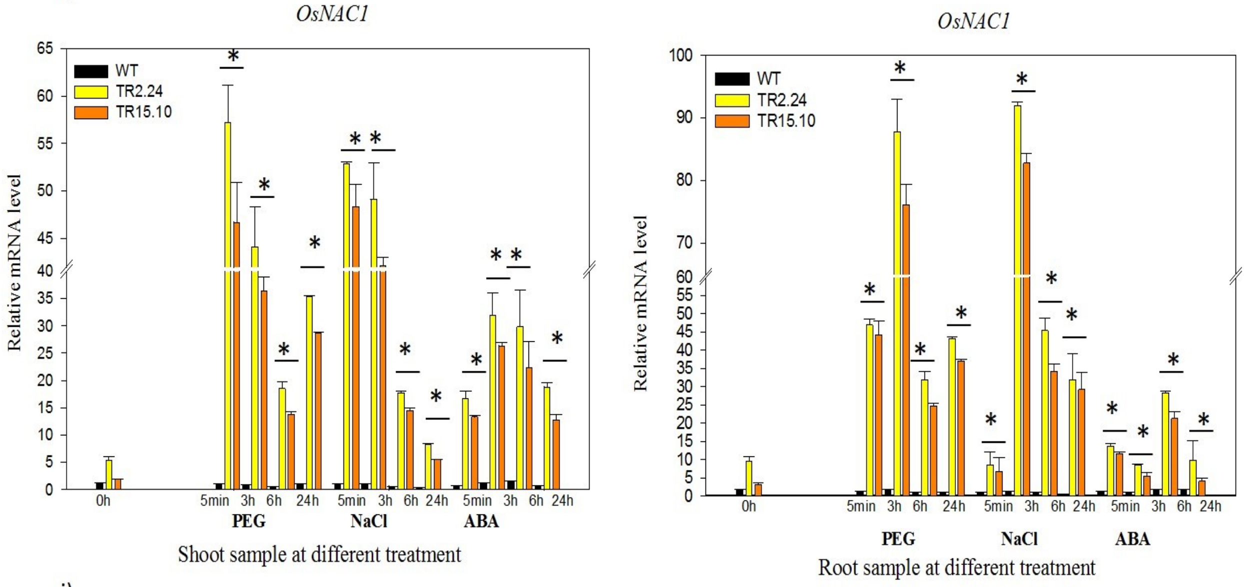

OsNAC1
WT
TR2.24
TR15.10
Relative mRNA level
0h
5min 3h 6h 24h 5min 3h 6h 24h 5min 3h 6h 24h
PEG NaCl ABA
Shoot sample at different treatment
OsNAC1
WT
TR2.24
TR15.10
Relative mRNA level
0h
5min 3h 6h 24h 5min 3h 6h 24h 5min 3h 6h 24h
PEG NaCl ABA
Root sample at different treatment

i)

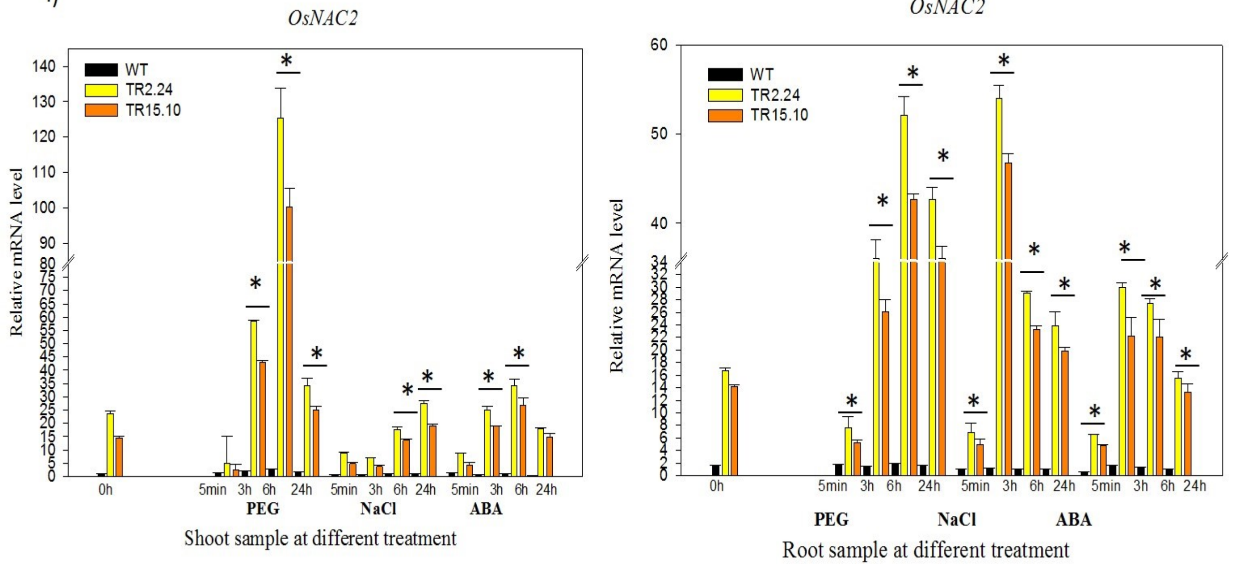

OsNAC2
WT
TR2.24
TR15.10
Relative mRNA level
0h
5min 3h 6h 24h 5min 3h 6h 24h 5min 3h 6h 24h
PEG NaCl ABA
Shoot sample at different treatment
OsNAC2
WT
TR2.24
TR15.10
Relative mRNA level
0h
5min 3h 6h 24h 5min 3h 6h 24h 5min 3h 6h 24h
PEG NaCl ABA
Root sample at different treatment

j)

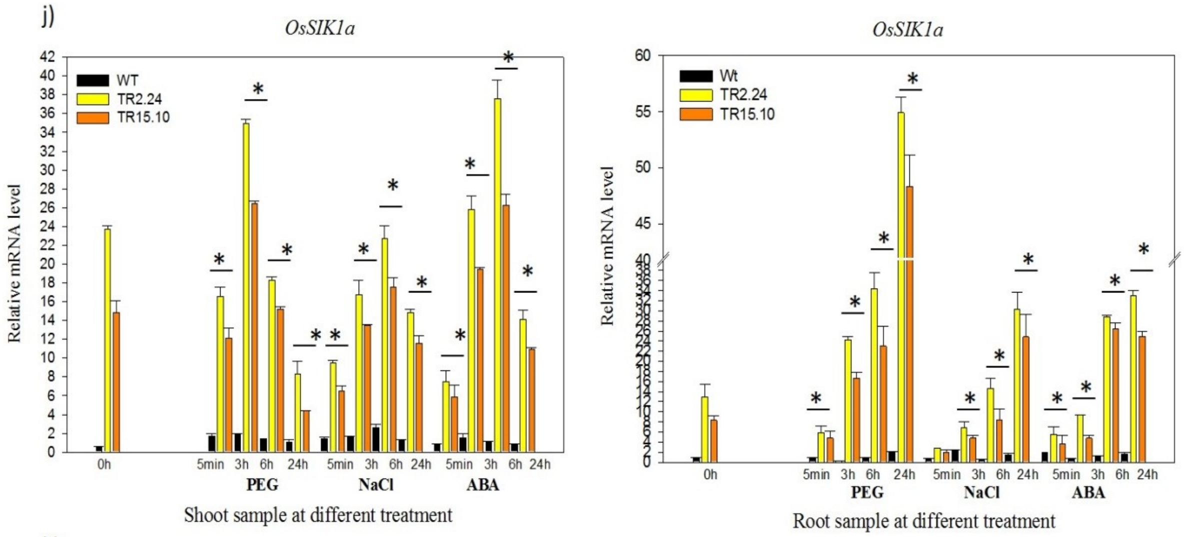
OsSIK1a
WT
TR2.24
TR15.10
Relative mRNA level
0h
5min 3h 6h 24h
PEG
NaCl
ABA
Shoot sample at different treatment
OsSIK1a
Wt
TR2.24
TR15.10
Relative mRNA level
Root sample at different treatment

k)

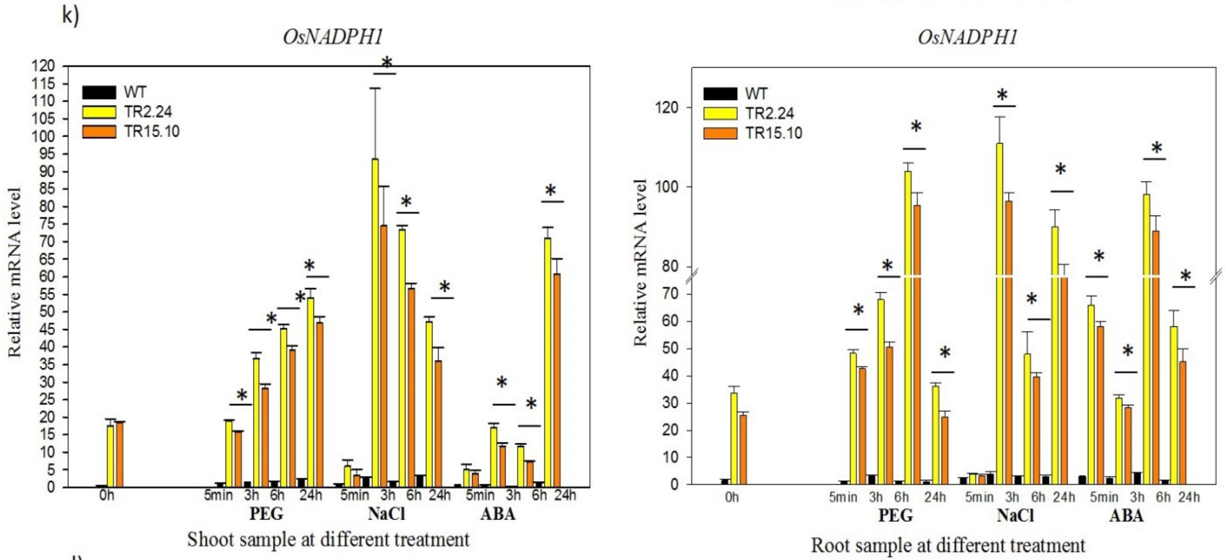
OsNADPH1
WT
TR2.24
TR15.10
Relative mRNA level
0h
5min 3h 6h 24h
PEG
NaCl
ABA
Shoot sample at different treatment
OsNADPH1
WT
TR2.24
TR15.10
Relative mRNA level
Root sample at different treatment

l)

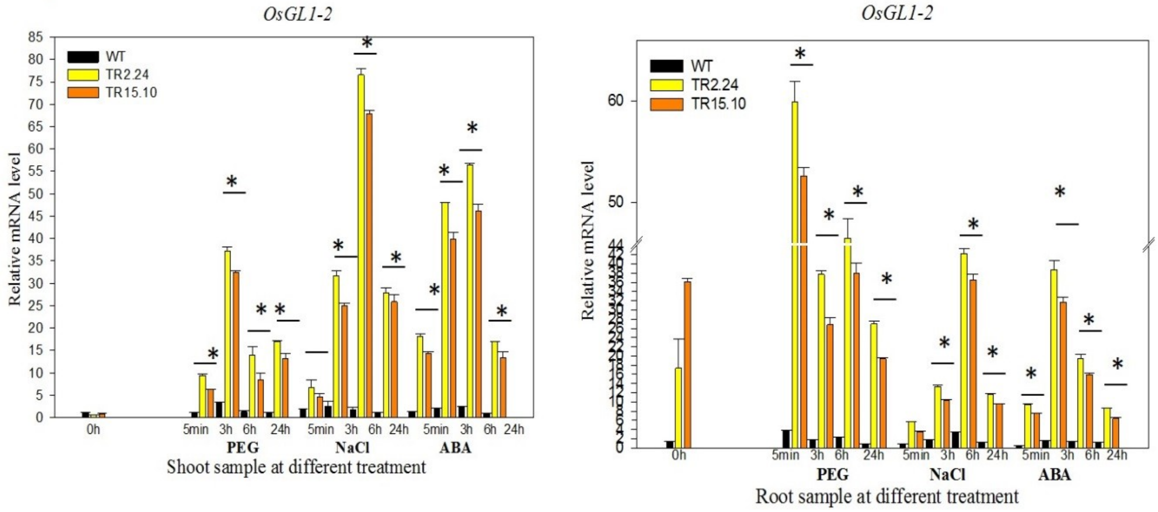
OsGL1-2
WT
TR2.24
TR15.10
Relative mRNA level
0h
5min 3h 6h 24h
PEG
NaCl
ABA
Shoot sample at different treatment
OsGL1-2
WT
TR2.24
TR15.10
Relative mRNA level
Root sample at different treatment

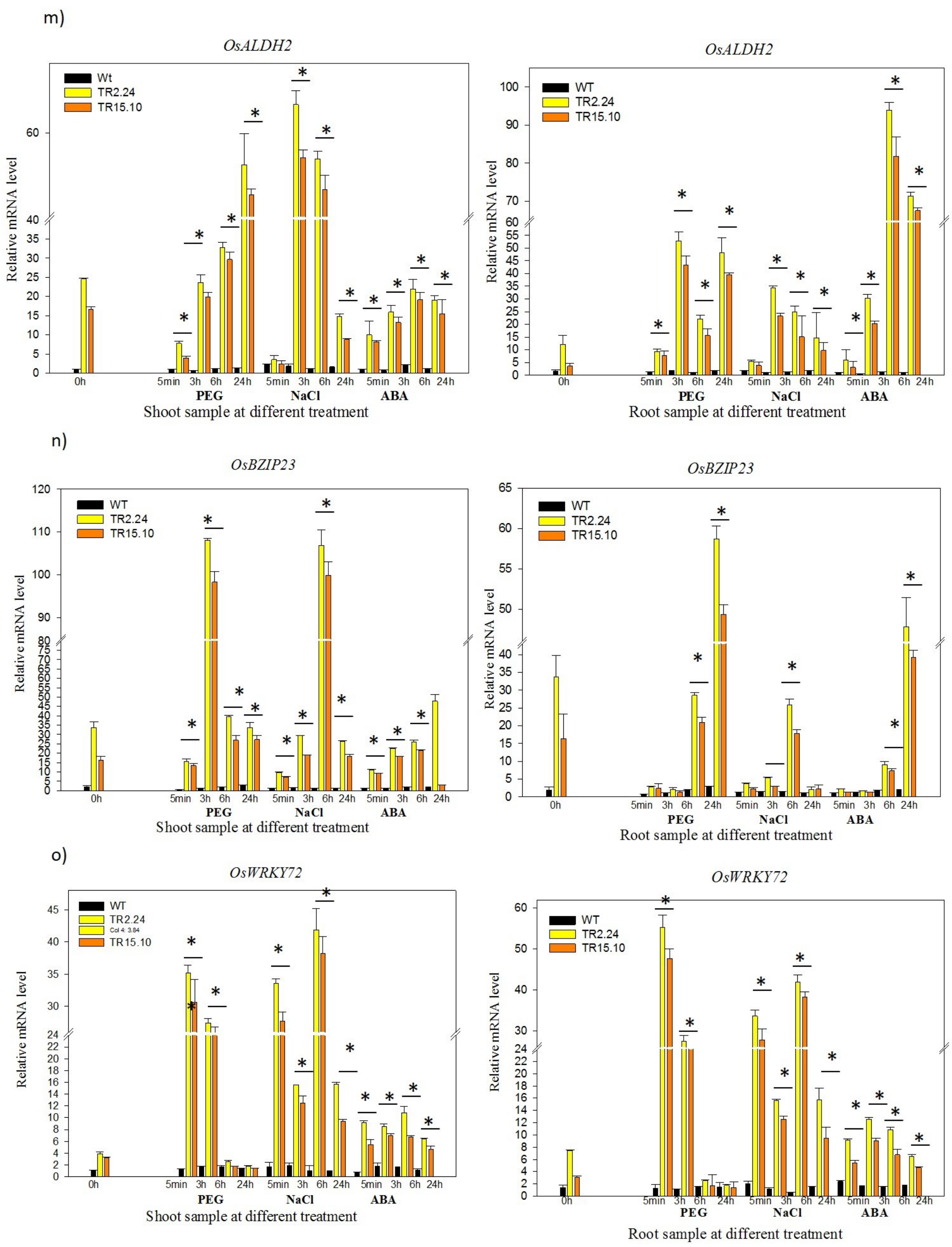

Fig. 4: 15 genes that specifically respond under stress conditions were studied in high expression lines of abiotic treated and untreated seedlings. Stress-specific genes such as (a)

OsPP2C (b) *OsSKIP1a* (c) *OsLEA3-1* (d) *OsTPP-1* (e) *OsAOX1a* (f) *OsDREB2B* (g) *OsDHODH1* (h) *OsNAC1* (i) *OsNAC2* (j) *OsSIK1a* (k) *OsNADPH1* (l) *GL1-2* (m) *OsALDH2* (n) *OsBZIP23* (o) *WRKY72* were studied in roots and shoots at different time intervals. The majority of the stress-specific genes were upregulated in both the high expression lines in untreated and treated conditions. The qRT-PCR data is a mean of three biological replicates. The relative expression was considered statistically significant at P value <0.05 (represented with asterisks) based on one-way ANOVA in all the analyzed genes

Supplementary Figure 5

In silico putative promoter analysis of a) *AtTOR* and b) *OsTOR*

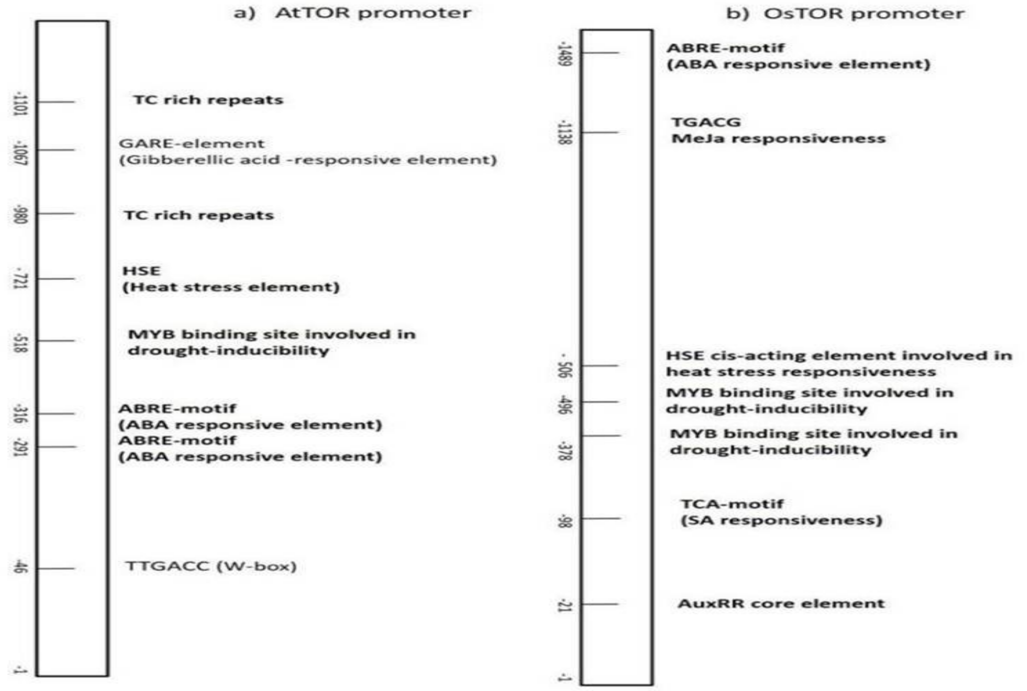

c) Phylogenetic tree representing TOR protein sequences from different organisms

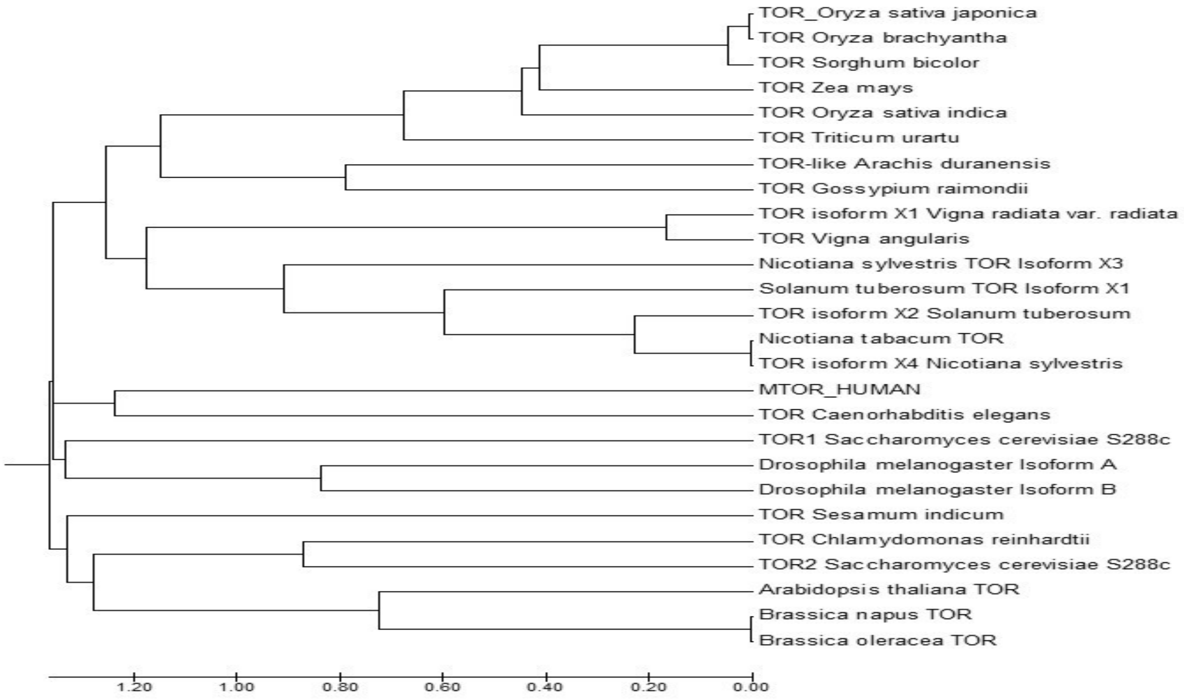

Fig. 5: Putative 1.5 kb promoter regions of (a) *AtTOR* and (b) *OsTOR* exhibited more than 45% similarity and contained many hormones and stress-responsive elements that likely interact with stress-specific transcription factors. (c) To check the protein similarity, sequences derived from 21 organisms were aligned in and a phylogenetic tree was developed.

The phylogenetic maps were generated using MEGA7 program by the text neighbor joining tree option with a boot strap value of 1000.
